# Combination HIV Prevention and HIV Incidence in Uganda

**DOI:** 10.1056/NEJMoa1702150

**Published:** 2017-11-30

**Authors:** Mary K Grabowski, David M Serwadda, Ronald H Gray, Gertrude Nakigozi, Godfrey Kigozi, Joseph Kagaayi, Robert Ssekubugu, Fred Nalugoda, Justin Lessler, Thomas Lutalo, Ronald Galiwango, Fred Makumbi, Xiangrong Kong, Donna Kabatesi, Stella T Alamo, Steven Wiersma, Nelson K Sewankambo, Aaron A R Tobian, Oliver Laeyendecker, Thomas C Quinn, Steven J Reynolds, Maria J Wawer, Larry W Chang

**Affiliations:** Department of Epidemiology, Johns Hopkins Bloomberg School of Public Health, Baltimore, MD; Rakai Health Sciences Program, Entebbe, Uganda; Department of Pathology, Johns Hopkins School of Medicine, Baltimore, MD; Rakai Health Sciences Program, Entebbe, Uganda; Makerere University School of Public Health, Kampala, Uganda; Department of Epidemiology, Johns Hopkins Bloomberg School of Public Health, Baltimore, MD; Rakai Health Sciences Program, Entebbe, Uganda; Rakai Health Sciences Program, Entebbe, Uganda; Rakai Health Sciences Program, Entebbe, Uganda; Rakai Health Sciences Program, Entebbe, Uganda; Rakai Health Sciences Program, Entebbe, Uganda; Rakai Health Sciences Program, Entebbe, Uganda; Department of Epidemiology, Johns Hopkins Bloomberg School of Public Health, Baltimore, MD; Rakai Health Sciences Program, Entebbe, Uganda; Rakai Health Sciences Program, Entebbe, Uganda; Rakai Health Sciences Program, Entebbe, Uganda; Makerere University School of Public Health, Kampala, Uganda; Department of Epidemiology, Johns Hopkins Bloomberg School of Public Health, Baltimore, MD; Centers for Disease Control, Uganda; Centers for Disease Control, Uganda; Centers for Disease Control, Uganda; Rakai Health Sciences Program, Entebbe, Uganda; Makerere University School of Medicine, Kampala, Uganda; Department of Epidemiology, Johns Hopkins Bloomberg School of Public Health, Baltimore, MD; Rakai Health Sciences Program, Entebbe, Uganda; Department of Pathology, Johns Hopkins School of Medicine, Baltimore, MD; Department of Epidemiology, Johns Hopkins Bloomberg School of Public Health, Baltimore, MD; Laboratory of Immunoregulation, Division of Intramural Research, National Institute for Allergy and Infectious Diseases, National Institutes of Health, Bethesda, MD; Division of Infectious Diseases, Department of Medicine, Johns Hopkins School of Medicine, Baltimore, MD; Department of Epidemiology, Johns Hopkins Bloomberg School of Public Health, Baltimore, MD; Laboratory of Immunoregulation, Division of Intramural Research, National Institute for Allergy and Infectious Diseases, National Institutes of Health, Bethesda, MD; Division of Infectious Diseases, Department of Medicine, Johns Hopkins School of Medicine, Baltimore, MD; Laboratory of Immunoregulation, Division of Intramural Research, National Institute for Allergy and Infectious Diseases, National Institutes of Health, Bethesda, MD; Division of Infectious Diseases, Department of Medicine, Johns Hopkins School of Medicine, Baltimore, MD; Department of Epidemiology, Johns Hopkins Bloomberg School of Public Health, Baltimore, MD; Rakai Health Sciences Program, Entebbe, Uganda; Department of Epidemiology, Johns Hopkins Bloomberg School of Public Health, Baltimore, MD; Rakai Health Sciences Program, Entebbe, Uganda; Division of Infectious Diseases, Department of Medicine, Johns Hopkins School of Medicine, Baltimore, MD

## Abstract

**BACKGROUND:**

To assess the impact of combination HIV prevention (CHP) on HIV incidence, we analyzed the association between HIV incidence and scale-up of antiretroviral therapy (ART) and medical male circumcision in Rakai, Uganda. Changes in population-level viral load suppression and sexual behaviors were also examined.

**METHODS:**

Between 1999 and 2016, data were collected through 12 surveys from 30 communities in the Rakai Community Cohort Study, an open population-based cohort of persons aged 15-49 years. We assessed HIV incidence trends based on observed seroconversion data, self-reported ART and male circumcision coverage, viral load suppression, and sexual behaviors.

**RESULTS:**

In total, 33,937 study participants contributed 103,011 person-visits (HIV prevalence ~13%). Follow-up of 17,870 HIV-negative persons contributed 94,427 person-years with 931 seroconversions. ART was introduced in 2004; by 2016 coverage was 69% (72% in women vs. 61% in men, p<0.001). HIV viral load suppression among all HIV-positive persons increased from 42% in 2009 to 75% by 2016 (p<0.001). Male circumcision coverage increased from 15% in 1999 to 59% by 2016 (p<0.001). Persons 15-19 years reporting n 71 ever having sex increased from 30% to 55% (p<0.0001). HIV incidence declined by 42% in 2016 relative to the pre-CHP period prior to 2010 (1.17/100 py to 0.66/100 py; adjIRR:0.58: 95%CI: 0.45-0.76); declines were greater in men (adjIRR=0.46; 95%CI: 0.29-0.73) than women (adjIRR=0.68, 95%CI: 0.50-0.94).

**CONCLUSIONS:**

In this longitudinal study, HIV incidence significantly declined with CHP scale-up, providing empiric evidence that HIV control interventions can have substantial population-level impact. However, additional efforts are needed to overcome gender disparities and achieve HIV elimination.

## INTRODUCTION

Combination HIV prevention (CHP) is the concurrent implementation of multiple interventions to reduce HIV incidence.^1^ Most CHP packages include antiretroviral therapy (ART) and medical male circumcision (MC), along with provision of HIV testing and counseling, condom promotion, and other behavioral interventions.^2^ CHP scale-up has been an intense focus of global health over the past decade.^3^

Modeling studies indicate that high coverage of ART and MC could substantially reduce HIV incidence to low-endemic levels,^4^ ^5^ and potentially even lead to its elimination.^6^ ^7^ However, the effectiveness of CHP remains uncertain due to challenges in increasing CHP coverage and in accurately measuring changes in population-level HIV incidence.^8^ ^9^ Demonstrating the population-level effectiveness of CHP is critical to understanding whether the current evidence98 based interventions are sufficient for HIV mitigation and to guide resource allocation.

While prior research from South Africa has shown that increasing community ART coverage reduces individual-level HIV risk, population-level HIV incidence declines were not demonstrated.^10^ ^11^ Other research from North America suggests that ART scale-up has reduced HIV incidence, but these studies relied on modeled incidence and sentinel surveillance data.^9^ ^12-14^ The “gold standard” for assessing HIV incidence is the longitudinal measurement of HIV seroconversions in a population-based cohort.^8^ ^9^ However, these studies are rare despite the urgency to demonstrate relationships between changes in CHP coverage and HIV incidence over time.^4^ ^5^ ^15^ To assess the impact of CHP on HIV incidence, we analyzed long-term trends in HIV incidence based on observed seroconversions and their associations with ART and MC scale-up, population-level viral load suppression, and sexual behaviors in Rakai, Uganda.

## METHODS

### Cohort Description

The Rakai Community Cohort Study (RCCS), conducted by the Rakai Health Sciences Program (RHSP), is an open, population-based, multi-community cohort of individuals aged 15-49 years.^16^ The RCCS is situated in Rakai District (population ~518,000) which is mostly rural with scattered trading centers.^17^ This study uses data from thirty RCCS communities which were continuously surveyed from April 6, 1999 to September 2, 2016 over a total of twelve surveys (Supplemental Fig.1).

To identify eligible participants, a household census enumerates all persons by gender, age, and duration of residence, regardless of whether they are present or currently absent. After the census, the RCCS surveys all present, age-eligible residents providing written informed consent. Participants are interviewed to assess demographics, sexual behaviors, ART use, and MC status. Venous blood is obtained for HIV testing at each survey (Supplemental RCCS laboratory methods). Funded by the U.S. President’s Emergency Plan for AIDS Relief (PEPFAR),^18^ CHP scale-up began in earnest in the mid-2000’s (Supplemental CHP scale-up).

### Statistical Analysis

CHP coverage was assessed using person-visit data at each survey with descriptive statistics and logistic regression. Specifically, ART coverage was defined as the proportion of all HIV positive participants who self-reported ART use, regardless of ART eligibility criteria, and was assessed overall and separately by gender. Self-reported ART use in the cohort has been validated previously by plasma detection of antiretroviral drugs showing a specificity and sensitivity of 99% (95%CI: 97-100%) and 77% (95%CI: 70-83%), respectively, with no differences by gender.^19^ MC coverage at a given visit was defined as the proportion of men who self-reported being circumcised. Self-reported circumcision status has been previously validated from clinical records with 100% specificity.^20^ Viral suppression was defined using a cutoff of 1000 copies/ml as per WHO recommendations.^21^

The unit of exposure for HIV incidence were person-intervals of follow-up between surveys in initially HIV-negative individuals who participated in at least two surveys. HIV incident cases were persons who tested HIV-seropositive for the first time with an HIV seronegative test result at the prior RCCS visit, allowing for up to one missed visit. Incident infections were assumed to occur at the mid-point of the interval and changes in HIV incidence per 100 person years (py) were estimated using Poisson multivariate regression with generalized estimating equations and an exchangeable correlation structure and were reported as incidence rate ratios (IRR) with 95% confidence intervals (CI).

To assess the impact of CHP, mean incidence at each visit interval after 2004 (6^th^ survey) was compared to mean HIV incidence over the entire period prior to ART and MC availability. The final multivariate model included individual-level information on demographics (gender, age, marital status, education) and sexual behaviors (sexual partners in the last year, sex with partners outside the community of residence, sex with non-marital partners, condom use and self-reported genital ulceration). A categorical term for community-level HIV prevalence was included to adjust for variation in exposure. Secondary analyses were stratified by gender and conducted separately for circumcised and uncircumcised men. HIV incidence and individual risk was also assessed in relation to community-level measures of ART and MC coverage and prevalence of HIV viremia (Supplemental statistical methods).

Sensitivity of results to both selective participation and loss to follow-up were evaluated using inverse probability weights (Supplemental statistical methods). To assess the potential impact of birth cohort effects on HIV incidence trends, a term for each five-year birth cohort was included in the multivariate model. HIV incidence was also assessed by gender for each five year age group.

## RESULTS

### Survey participation

Table 1 shows eligibility and participation summary statistics for the twelve surveys. Overall, 33,937 individual participants contributed 103,011 person-visits, including an incidence cohort of 17,870 initially HIV-negative persons followed for 94,427 person-years. The mean participation rate among all eligible persons censused was 64% and did not vary substantially between surveys (range: 59%-67%); however, reasons for non-participation and study drop-out (e.g. refusal, travel) changed over time (Supplemental Tables.1a-c, 2a-c). The proportion of individuals who refused participation steadily declined from 21% to 0.5% over the analysis period, whereas the proportions absent due to work or school increased from 18% to 31%. The most common reasons for loss to follow-up were out-migration from study communities (ranging from 42-63% of losses) and travel for work or school (ranging from 25-33% of losses).

**Table 1.** Summary of eligibility, participation and follow-up in the RCCS by survey round, 1999-2016

Survey	Interview Date	Census Eligibleα	Eligible and present for surveyβ	Percent eligible who participated in surveyγ	Percent eligible and present who participated in survey	HIV-negative participants eligible for incidence cohort*	Percent of eligible HIV-negative participants who outmigrated prior to the subsequent survey	Incidence cohort	Percent of age-eligible HIV-negative participants followed	Percent of age and resident eligible HIV-negative participants followed**	Years since prior survey visit
	Median (range)	no.	no.	Percent (no.)	Percent	no.	Percent (no.)	no.	Percent	Percent	median (IQR)
1	Oct.1999 (Apr.1999-Feb.2000)	9869	8125	61% (5992)	74%	-	-	-	-	-	-
2	Oct.2000 (Feb. 2000-Feb.2001)	10448	8567	64% (6732)	79%	5183	11% (546)	3760	73%	93%	1.0 (1.0,1.0)
3	Jan.2002 (Apr.2001-May.2002)	11316	9176	65% (7340)	80%	7277	23% (1677)	4540	62%	82%	1.3 (1.1,1.3)
4	Apr.2003 (Jul.2002-Aug.2003)	11436	8603	60% (6856)	80%	7905	27% (2167)	4555	58%	80%	1.2 (1.2,1.3)
5	Jul.2004 (Sep.2003-Nov.2004)	11860	8436	59% (7038)	83%	8014	28% (2206)	4693	59%	81%	1.3 (1.2,1.3)
6	Jan.2006 (Feb.2005-Jun.2006)	12528	9137	65% (8097)	89%	7768	28% (2159)	4867	63%	87%	1.5 (1.4,1.6)
7	Oct.2007 (Aug.2006-Jun.2008)	13636	9130	63% (8645)	95%	8624	30% (2585)	5001	58%	83%	1.7 (1.6,1.8)
8	Jul.2009 (Jun.2008-Dec.2009)	13293	9009	65% (8691)	96%	9679	30% (2952)	5611	58%	84%	1.7 (1.6,1.8)
9	Jan.2011 (Jan.2010-Jun.2011)	14629	9949	66% (9643)	97%	9686	30% (2894)	5742	59%	85%	1.6 (1.6,1.6)
10	Jun.2012 (Aug.2011-May.2013)	16007	10846	66% (10588)	98%	10300	29% (3032)	6176	60%	85%	1.6 (1.5,1.7)
11	Jul.2014 (Jul.2013-Jan.2015)	17477	11566	65% (11379)	98%	11419	34% (3875)	6277	55%	83%	2.0 (1.9,2.1)
12	Jan.2016 (Jan.2015-Sep.2016)	18065	12308	66% (12010)	98%	12908	31% (4017)	7122	55%	80%	1.6 (1.4,2.0)

α Residents aged 15-19 in the census.

β Eligible census population present at time of survey,

γ Eligible census population present and participated in survey.

* Includes all age-eligible HIV-negative participants from prior survey and any HIV-negative participants from two surveys prior if participant was absent at the most recent survey.

** Calculation excludes HIV-negative persons who out-migrated prior to survey.

**Table 2:** HIV incidence and unadjusted and adjusted incidence rate ratios comparing HIV incidence in each visit interval during combination HIV prevention (CHP) scale-up to mean HIV incidence in the entire period prior to scale-up. IRR=Incidence Rate Ratio; adjIRR=Adjusted incidence rate ratio; Final adjusted model included age, gender (full cohort only), marital status, level of education, number of sexual partners in past year, sex with partners outside community, self-reported genital ulcer disease, condom use with casual partners, community residence type (trading, agrarian), and community HIV prevalence.

HIV incidence Cohort (N=17,780)							
Survey(s)	Incident HIV cases	person-years	HIV incidence per 100 py (95%CI)	IRR (95%CI)	p-value	adjIRR (95% CI)	p-value
Pre-CHP (2-5)	254	21765	1.17 (1.03,1.32)	Ref.	-	Ref.	-
Jan.2006 (6)	86	7773	1.11 (0.89,1.36)	0.95 (0.74,1.21)	0.66	0.94 (0.73,1.2)	0.61
Oct.2007 (7)	105	8769	1.2 (0.98,1.44)	1.02 (0.82,1.29)	0.84	1.00 (0.79,1.26)	0.99
Jul.2009 (8)	125	10201	1.23 (1.02,1.45)	1.05 (0.85,1.3)	0.67	0.95 (0.76,1.18)	0.62
Jan.2011 (9)	105	9815	1.07 (0.88,1.29)	0.91 (0.73,1.15)	0.44	0.94 (0.74,1.19)	0.60
Jun.2012 (10)	86	10352	0.83 (0.67,1.02)	0.71 (0.55,0.91)	0.006	0.72 (0.56,0.93)	0.012
Jul.2014 (11)	87	13159	0.66 (0.53,0.81)	0.56 (0.44,0.72)	<0.001	0.60 (0.47,0.78)	<0.001
Jan.2016 (12)	83	12593	0.66 (0.53,0.81)	0.56 (0.44,0.72)	<0.001	0.58 (0.45,0.76)	<0.001

Women (N=9,709)							
Survey(s)	Incident HIV cases	person-years	HIV incidence per 100 py (95%CI)	IRR (95%CI)	p-value	adjIRR (95% CI)	p-value
Pre-CHP (2-5)	145	12409	1.17 (0.99,1.37)	Ref.	-	Ref.	-
Jan.2006 (6)	50	4425	1.13 (0.84,1.47)	0.97 (0.7,1.33)	0.84	0.98 (0.71,1.35)	0.90
Oct.2007 (7)	65	4978	1.31 (1.01,1.65)	1.12 (0.83,1.5)	0.46	1.10 (0.82,1.48)	0.52
Jul.2009 (8)	67	5610	1.19 (0.93,1.5)	1.02 (0.76,1.36)	0.89	0.91 (0.68,1.23)	0.55
Jan.2011 (9)	61	5319	1.15 (0.88,1.46)	0.98 (0.73,1.32)	0.90	0.98 (0.72,1.33)	0.89
Jun.2012 (10)	50	5587	0.89 (0.67,1.17)	0.77 (0.56,1.05)	0.10	0.75 (0.54,1.05)	0.096
Jul.2014 (11)	55	7090	0.78 (0.59,1)	0.66 (0.49,0.90)	0.010	0.65 (0.47,0.91)	0.012
Jan.2016 (12)	56	6689	0.84 (0.64,1.08)	0.72 (0.53,0.97)	0.034	0.68 (0.50,0.94)	0.021

Men (N=8,161)							
Survey(s)	Incident HIV cases	person-years	HIV incidence per 100 py (95%CI)	IRR (95%CI)	p-value	adjIRR (95% CI)	p-value
Pre-CHP (2-5)	109	9356	1.17 (0.96,1.4)	Ref.	-	Ref.	-
6	36	3348	1.08 (0.76,1.47)	0.92 (0.63,1.34)	0.661	0.90 (0.61,1.33)	0.60
7	40	3791	1.06 (0.76,1.42)	0.90 (0.63,1.29)	0.572	0.89 (0.62,1.3)	0.56
8	58	4591	1.26 (0.97,1.62)	1.08 (0.78,1.48)	0.641	1.02 (0.73,1.42)	0.93
9	44	4497	0.98 (0.72,1.3)	0.83 (0.59,1.18)	0.309	0.92 (0.63,1.33)	0.64
10	36	4765	0.76 (0.53,1.03)	0.64 (0.44,0.94)	0.022	0.70 (0.47,1.05)	0.083
11	32	6069	0.53 (0.37,0.73)	0.45 (0.3,0.67)	<0.001	0.54 (0.36,0.83)	0.005
12	27	5904	0.46 (0.31,0.65)	0.39 (0.25,0.59)	<0.001	0.46 (0.29,0.73)	0.001

Uncircumcised men only (N=5,660)							
Survey(s)	Incident HIV cases	person-years	HIV incidence per 100 py (95%CI)	IRR (95%CI)	p-value	adjIRR (95% CI)	p-value
Pre-CHP (2-5)	94	7773	1.21 (0.98,1.47)	Ref.	-	Ref.	-
Jan.2006 (6)	30	2456	1.22 (0.83,1.71)	1.01 (0.67,1.52)	0.98	0.96 (0.63,1.46)	0.85
Oct.2007 (7)	31	2590	1.2 (0.82,1.67)	0.98 (0.66,1.48)	0.94	0.92 (0.61,1.4)	0.70
Jul.2009 (8)	40	2927	1.37 (0.99,1.83)	1.12 (0.78,1.63)	0.54	1.00 (0.68,1.47)	0.99
Jan.2011 (9)	32	2571	1.24 (0.86,1.73)	1.02 (0.68,1.53)	0.92	1.00 (0.67,1.51)	0.98
Jun.2012 (10)	24	2493	0.96 (0.63,1.4)	0.79 (0.50,1.24)	0.30	0.77 (0.49,1.22)	0.27
Jul.2014 (11)	17	2779	0.61 (0.36,0.95)	0.50 (0.30,0.84)	0.009	0.46 (0.26,0.81)	0.007
Jan.2016 (12)	15	2303	0.65 (0.37,1.04)	0.53 (0.31,0.92)	0.024	0.51 (0.29,0.88)	0.016

Participation and follow-up rates were significantly lower among younger individuals, men, and persons living in trading centers, but these associations were stable over time. Individuals with high-risk sexual behaviors were somewhat more likely to be lost to follow-up but this was also constant over time. (Supplemental Figs.2-4). The population growth rate, calculated from the censused resident population irrespective of age, was 3.4% per year. 

### Temporal trends in sexual behaviors

**Figure 1.**
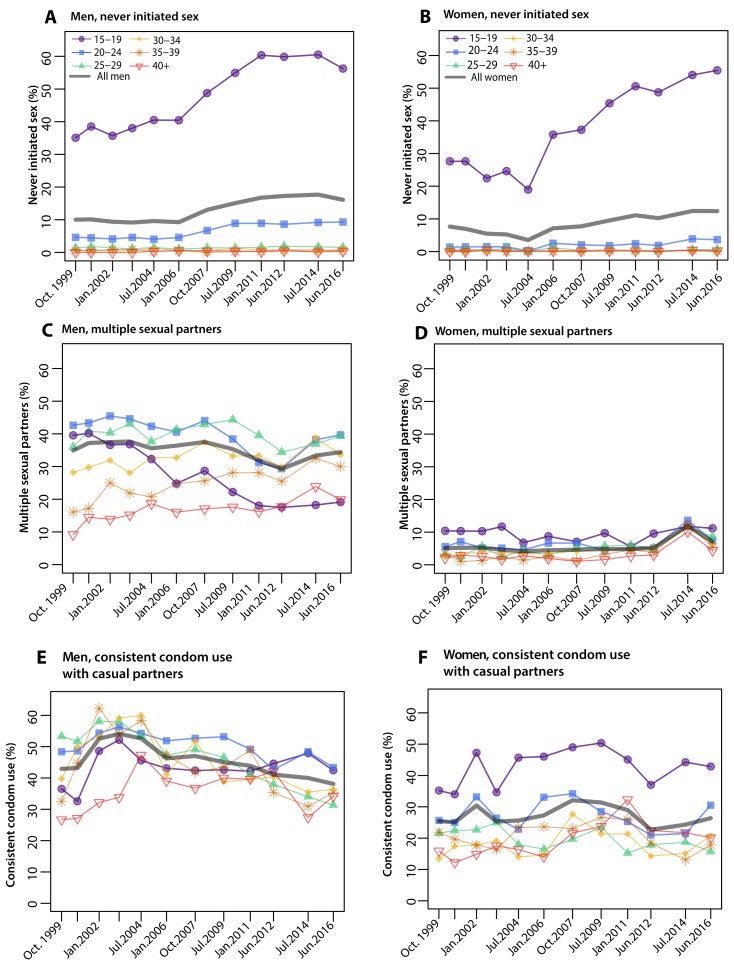
Sexual Behaviors in the Rakai Community Cohort Study, 1999-2016. Figure shows proportion of HIV-negative men and women by age-group and overall ages reporting the following sexual behaviors A-B) never initiating sex (i.e. delayed sexual debut), C-D) multiple sexual partnerships among sexually active persons, and E-F) consistent condom use among those reporting casual (i.e. non-marital) sexual partnerships. The most substantial changes in sexual behaviors occurred among adolescent men and women aged 15-19 years reporting never initiating sex and adolescent men reporting multiple partnerships.

Figure 1 shows age-specific sexual behaviors by survey for HIV-negative men and women. The most substantive changes in sexual behaviors were in adolescents aged 15-19, among whom the proportion self-reporting no initiation of sex increased from 30% in 1999 to 55% in 2016 (p<0.0001) overall, and from 35% (n=194/553) to 56% (n=679/1207) in men, and 28% (n=209/757) to 55% (n=646/1165) over the same time period (p<0.001 for both). Adolescent men who initiated sex were also significantly less likely to report multiple sexual partners in the last survey (40% in 1999 versus 19% in 2016, p<0.001). There were no substantial changes in female multiple partnerships. Overall ages, levels of self-reported condom use with casual partners remained largely unchanged (Figures 1E and 1F).

### Scale up of biomedical HIV interventions and changes in population HIV viral load

**Figure 2.**
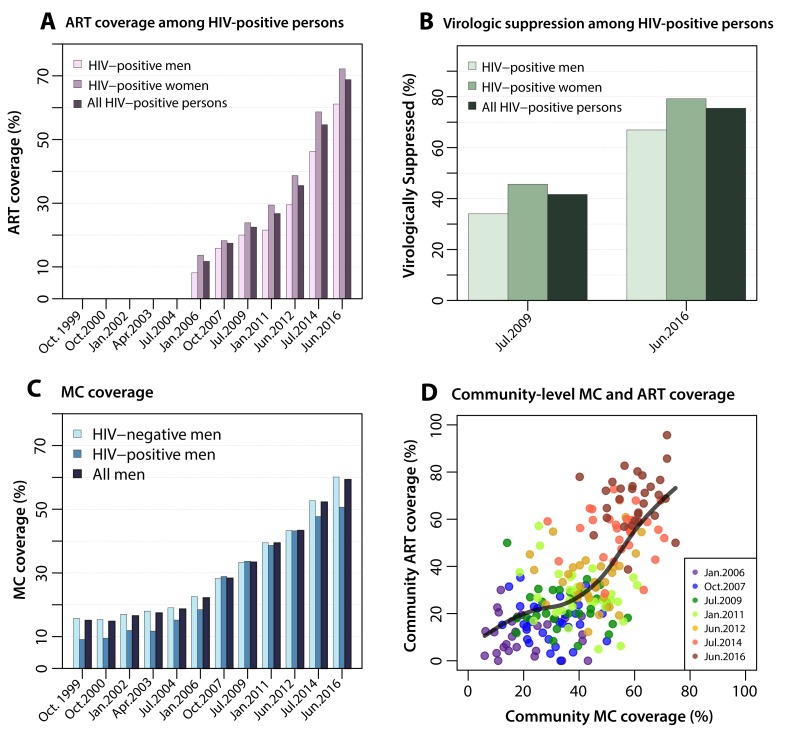
Scale-up of antiretroviral therapy, viral suppression in HIV-positive participants and male circumcision, 1999-2016. **2A** shows scale-up of ART coverage measured by selfreport in men, women and all HIV-positive RCCS participants beginning in 2006. Figure 2B show the proportion of all HIV-positive persons by gender and overall virologically suppressed (<1000 HIV copies/ml) in 2009 and 2016. **2C** shows scale-up of MC coverage in men irrespective of religion by HIV status and overall beginning in 2004. **2D** shows community-level MC coverage vs. community-level ART coverage for all 30 communities at each survey during CHP scale-up. A smoothing-spline was fit to the smooth curve to assess trend. Scale-up of interventions occurred simultaneously and increased significantly in all communities.

The scale-up of biomedical HIV prevention interventions is shown in Figure 2. Self-reported ART use among all HIV-positive persons increased from 12% in 2006 to 69% in 2016 (p<0.001). ART coverage was consistently higher among women (p<0.001); however, the proportional increase in coverage was similar in both genders. By 2016, 61% of HIV-positive men (n=285/465) and 72% (n=766/1060) of women self-reported ART use (Supplemental Table A). ART coverage was highest among older age groups in all surveys (Supplemental Fig.5).

HIV viral load assays were obtained for 96% (1115/1160) of HIV-positive participants in 2009 and for 99.9% (1525/1526) of HIV-positive participants in 2016. Viral load suppression (<1000 cps mL) among those self-reporting ART use was 94% (n=1228/1312) and did not differ by gender (p=0.382) or survey visit (p=0.525). HIV viral load suppression in all HIV-positive participants increased concomitant with increasing ART coverage. By 2016, 75% (n=1151/1526) of all HIV-positive persons, regardless of whether or not they reported ART use, were virally suppressed compared with 42% (n=464/1115) in 2009 (p<0.001) (Figure 2B).

Population coverage of MC also significantly increased from 15% (n=374/2518) in 1999 to 59% (n=3177/5361) in 2016 among all men (p<0.001) (Figure 2C), and from 3.5% (n=77/2217) to 53% (n=2492/4666, p<0.001) among non-Muslim men who are not traditionally circumcised at birth. MC coverage increased among both HIV-positive and HIV-negative men with highest coverage in younger men (Supplemental Table 3B, Supplemental Fig.5).

Scale-up of ART and MC occurred concurrently in all communities (Figure 2D) and by 2016 were high in all 30 RCCS communities: median community-level ART coverage was 70% (IQR: 61-75) and median community-level MC coverage was 61% (IQR:55-65%).

### Changes in HIV incidence over time

**Figure 3.**
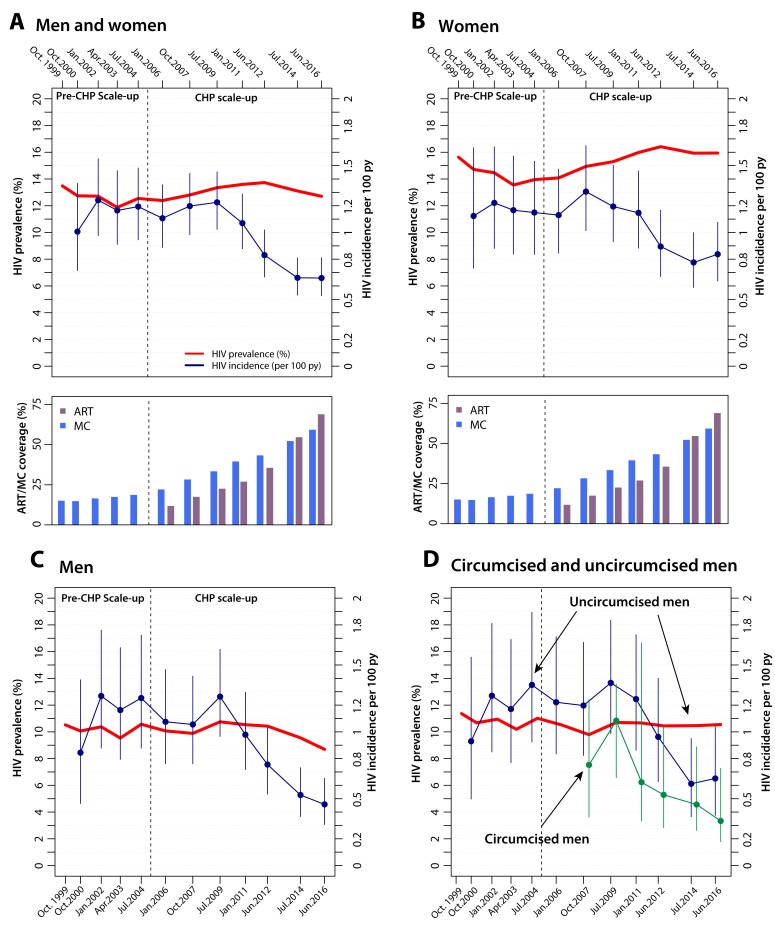
HIV incidence and prevalence trends in the Rakai Community Cohort Study, 1996-2016. Trends in HIV incidence and prevalence over the analysis period among all initially HIV-negative men and women in the incidence cohort **(3A)**, women only **(3B)**, men only **(3C)**, and in men by circumcision status **(3D)**. HIV incidence is only shown for circumcised men ginning in 2007 after the WHO recommendation for MC for HIV-negative men for HIV prevention. HIV prevalence is shown in red and HIV incidence and 95% CI for each visit interval are shown in blue (green for circumcised men). The ART and MC coverage plots are also included to show the temporal association between scale-up of CHP and declines in HIV incidence.

Figure 3 shows HIV incidence in the whole population, women, men, and circumcised and uncircumcised men. HIV incidence remained stable prior to CHP scale-up and began to significantly decline in 2012 (Fig. 3, Supplementary Table.4a-e). In 2016, mean HIV incidence declined by 42% from 1.17 per 100 py prior to CHP to 0.66 per 100 py (IRR=0.56, 95%CI: 0.44- 0.72; adjIRR=0.58; 0.45-0.76). The same incidence trends were observed when restricting analyses to sexually active adults and individuals over the age of 20 years (Supplementary Tables.4,5).

Declines in incidence were greater in men (adjIRR=0.46; 95%CI: 0.29-0.73) than in women (adjIRR=0.68, 95%CI: 0.50-0.94). HIV incidence was lower in circumcised compared to uncircumcised men (adjIRR=0.61: 95%CI: 0.48-0.79), but incidence declined significantly in both circumcised men (adjIRR=0.43; 95%CI: 0.19-0.99) and uncircumcised men by 2016 (adjIRR: 0.51, 95%CI: 0.29-0.88) (Fig.3, Supplementary Tables.4b-e).

There were HIV incidence declines among the majority of male and female age groups, and among both genders residing in trading and agrarian communities (Supplementary Fig.6-7). In sensitivity analyses, inclusion of birth cohort or inverse probability weights for selective participation and follow-up did not change inferences (Supplementary Tables 7-8).

Though CHP coverage concurrently increased across RCCS communities (Fig. 2D., Supplemental Fig.7-8), we also assessed HIV incidence and individual-level HIV risk as functions of ART coverage, population prevalence of viremia, and MC coverage at the community-level. These analyses showed declining incidence and lower individual-level risk with increasing community ART and MC coverage and declining population viremia (Supplemental Fig.9-11).

## DISCUSSION

In this study, HIV incidence significantly declined with CHP scale-up, providing some of the first empiric evidence that CHP can have substantial population-level impact. The declines in HIV incidence are likely due to ART and MC scale-up; reduced sexual activity in late adolescence may also have contributed. HIV incidence declined less in women compared to men, suggesting that the combined direct effects of MC and indirect effects of female ART use differentially benefited men. Additional efforts are needed to avert new infections in women such as further scale-up of ART in men and potentially introducing new primary prevention interventions (e.g. Pre-exposure Prophylaxis or PrEP).

We previously found that community-levels of MC and female ART at modest coverage levels were associated with lower community HIV incidence in males.^22^ A study in rural South Africa reported lower risk of individual-level HIV acquisition associated with higher rates of ART coverage, but that study did not assess temporal declines in incidence or MC coverage.^10^ Our finding of a 42% reduction in HIV incidence to 0.66/100py is substantial, but still well above the 0.1/100py incidence rate estimated as the threshold for HIV elimination.^6^ ^23^

From 2009 to 2016, the proportion of HIV-positive persons with viral suppression increased by 46%, suggesting that HIV viral suppression via ART likely reduced HIV exposure to uninfected opposite sex partners, consistent with other studies.^12^ ^13^ ^24-26^ By 2016, the rate of virologic suppression among HIV-positive persons was 75%, meeting the 2020 goal of the UNAIDS 90-90-90 initiative which modeling suggests could end the HIV epidemic by 2030.^27^ Our results demonstrate that ambitious ART scale-up goals can be achieved. Similar viral suppression results have been reported in Botswana (71%), although beneficial effects on HIV incidence rates in Botswana have not yet been reported.^28^ ^29^

MC coverage steadily increased to 59% by 2016, but remained below UNAIDS targets of 80% coverage.^30^ Scale up of ART and MC were highly correlated (Fig. 2D) so it is difficult to disaggregate their effects. Nevertheless, we attempted to address this issue empirically by assessing HIV incidence trends separately in men and women and in uncircumcised and circumcised men. Prior mathematical modeling studies suggest that there are substantial, long term indirect effects of MC on both female partner HIV incidence and in uncircumcised men; however, these benefits are unlikely to be realized until at least a decade after HIV prevalence declines resulting from direct effects of MC.^31^ Therefore, the significant reductions of HIV incidence in women and uncircumcised men observed in this study most likely result from the population-level impact of increasing ART coverage on HIV incidence. Notably, circumcised men had the sharpest declines in HIV incidence, nearly twice as great as uncircumcised men, likely because they benefit from the direct protective effect of MC and from the indirect effect of female partners on ART. In comparison, women and uncircumcised men had more moderate declines in incidence, likely because they largely benefit from indirect reduced exposure afforded by their partner’s ART use. Rates of ART use were lower in HIV-positive men, which would further attenuate benefits for women.^31^

Statistically significant HIV incidence declines were first observed in 2012 when ART and MC coverage levels reached 36% and 43%, respectively. It would be tempting to conclude that these coverage levels represent threshold effects, but because interventions were scaled concurrently and the impact of interventions may be delayed, we cannot reliably make such inferences from these empiric data alone. Defining intervention thresholds would also depend on the proportion of infections introduced from outside the population of interest, a quantity which likely varies across settings.

We found reductions in sexual activity in both males and females aged 15-19. Prior RHSP studies showed a decline in HIV incidence among 15-19 year-old girls associated with factors such as delayed sexual debut, coincident with increased school enrollment.^32^ However, this age group represents a small fraction of all incident HIV infections in the RCCS with limited behavioral changes in older age groups suggesting its impact on population HIV incidence are likely modest. Of note, there were no significant changes in condom use in any age group which is sobering given many years of condom promotion and provision.

This observational study meets almost all of Hill’s criteria for causality including a strong temporal association between CHP scale-up and HIV incidence declines, a dose-response relationship (i.e., greater declines in HIV incidence with increasing CHP coverage), consistency with prior studies of ART and MC, and biological plausibility.^33^ However, the study has a number of limitations. ART and MC coverage, and sexual behaviors were self-reported and may be subject to social desirability and other biases. However, there are no clear indications that any biases changed over time, and self-reported ART has been validated with high specificity in this population.^19^ Viral load testing was conducted on stored sera which may be subject to RNA degradation over time, potentially resulting in overestimation of viral suppression in the earlier survey and an underestimation of the magnitude of viral suppression over time.^34^ While RCCS has relatively high participation rates compared to other African population-based cohorts, there was substantial mobility which reduced participation and follow-up.^35^ ^36^ However, participation rates among those present in the community increased over time, and sensitivity analyses to assess potential selection bias did not change our inferences.

An important consideration is whether these CHP coverage and HIV incidence results can be generalized. RCCS demographic and behavioral data are largely consistent with Demographic and Health Surveys in the region.^37^ RCCS is an open population-based cohort with extensive in and out-migration which likely minimized, though did not eliminate, potential Hawthorne effects of repeat observations. RHSP has conducted CHP intervention and prevention studies which may have increased ART and MC coverage.^38-40^ All RCCS participants are offered HIV testing services resulting in high coverage (98% in 2015). Although conditions in Rakai may have been favorable for rapidly scaling CHP services, the impact of these interventions on population-level HIV incidence provides proof of concept and should be generalizable. Indeed, data from the Uganda Ministry of Health’s National AIDS Control Program data indicates dramatic scale-up of CHP was also occurring nationally: ART and MC coverage were 68% and 54%, respectively, in 2016 (Steven Wiersma, personal communication, 2017).

In summary, data from this longitudinal cohort in Rakai, Uganda show a 42% decline in HIV incidence associated with CHP, providing evidence that HIV control efforts can have a substantial population-level impact. Differential declines in HIV incidence by gender indicate a need for strengthening CHP efforts to benefit women, including improving ART coverage in men and consideration of newer, primary prevention interventions such as PrEP. Intensification of CHP efforts for both women and men including key underserved populations such as migrants, as well as long-term surveillance, are needed to determine whether HIV incidence can be further reduced to the levels necessary for elimination.

## Supplementary Material

Supplementary Appendix
