# Supplementary material for: Combination HIV Prevention and HIV Incidence in Uganda
Source: N Engl J Med. Author manuscript; Available in PMC 2017 Nov 30. (PMC5627523; doi:10.1056/NEJMoa1702150)
Supplement: Supplementary Appendix [file NEJMoa1702150_Grabowski_Supplement.pdf]

SUPPLEMENTAL APPENDIX

Combination HIV Prevention and HIV Incidence in Uganda

M.K. Grabowski, D.M. Serwadda, R.H. Gray, G. Nakigozi, G. Kigozi, J. Kagaayi, R. Ssekubugu, F. Nalugoda, J. Lessler, T. Lutalo, R.Galiwango, F. Makumbi, X. Kong, D. Kabatesi, S. T. Alamo, S. Wiersma, N. K. Sewankambo, A.A.R. Tobian, O. Laeyendecker, T.C. Quinn, S.J. Reynolds, M.J. Wawer, and L.W. Chang, for the Rakai Health Sciences Program

Table of Contents

Acknowledgment of contributors 3

RCCS Ethics Approval 4

History of the RCCS and study design 4

Combination HIV prevention Scale-up 4-5

RCCS laboratory methods 5

History of RCCS HIV testing algorithms 6-7

Summary and description of variables included in primary multivariate regression analyses .. 8-13

Analysis of HIV incidence and community ART coverage, community prevalence of viremia, and community MC coverage 14

Estimation stabilized inverse probability weights methods 14

Figure S1. Map of Rakai region and 30 continuously surveyed RCCS study communities 15

Table S1a-c. Summary of RCCS participation by survey round 16-17

Table S2a-c. Summary of reasons for loss to follow-up.... 17-18

Figure S2. Summary of RCCS participation by age, gender, and community-type 19

Figure S3. Summary of loss to follow-up by age, gender, and community-type 20

Figure S4. Sexual behaviors among HIV-negative participants 21

Table S3A. ART coverage by survey and gender....22

Table S3B. MC coverage by survey and gender....23

Figure S5. ART and male circumcision coverage by age and gender.... 24-25

Table S4A. HIV incidence among all participants comparing during and prior to CHP 26-27

Table S4B. HIV incidence among women comparing during and prior to CHP 28-29

Table S4C. HIV incidence among men comparing during and prior to CHP 29-30

Table S4D. HIV incidence among uncircumcised men comparing during and prior to CHP. 30-31

Table S3E. HIV incidence among circumcised men comparing during and prior to CHP 32-33

Table S5. HIV incidence among 20 years and older comparing during and prior to CHP 34

Table S6. HIV incidence among sexually active persons comparing during and prior to CHP....34

Table S7. HIV incidence in each visit interval adjusted for birth cohort 36

Table S8. Inverse probability weighted HIV incidence rate ratios....36

Figure S5. Histogram of stabilized inverse probability weights....37

Figure S6A. HIV incidence among women by age-group....38

Figure S6B. HIV incidence among men by age-group....39

Figure S6A. HIV incidence by gender and community-type 40

Figure S7. Scale-up of ART and declining population viremia in RCCS communities....41

Figure S8. Scale-up of MC in RCCS communities 42

Figure S9. HIV incidence and community ART coverage....43

Figure S10. HIV incidence and community prevalence of viremia....44

Figure S11. HIV incidence and community MC coverage....45

Acknowledgment of Contributors

Principle Investigators of the Rakai Community Cohort Study:
David Serwadda
Ronald H. Gray
Maria J. Wawer

Corresponding Author:
Mary K. Grabowski

All authors provided input on the study design and contributed to writing of the final manuscript. Dr. Grabowski, Dr. Chang, and Dr. Wawer wrote the first draft of the manuscript. Drs. Grabowski and Chang had full access to the data and takes responsibility for the data presented. Dr. Serwadda, Dr. Nalugoda, Dr. Sewankambo, Mr. Ssekubugu, Dr. Kagaayi, Dr. Kigozi, Dr. Nakigozi, Dr. Reynolds, Dr. Gray, and Dr. Wawer supervised RCCS data collection. Dr. Grabowski and Dr. Lessler performed the data analysis. Mr. Lutalo and Dr. Makumbi performed data management and with Dr. Kong provided valuable input on data analysis. Dr. Nalugoda, Dr. Kagaayi, Dr. Kigozi, Dr. Nakigozi, Dr. Alamo, Dr. Wiersma, and Dr. Kabetesi supervised Combination HIV prevention scale-up, evaluation and monitoring of HIV prevention and treatment services in the Rakai District. Dr. Galiwango, Dr. Tobian, Dr. Reynolds, Dr. Laeyendecker and Dr. Quinn managed laboratory testing and storage of biological specimens.

Rakai Health Sciences Program Study Team Contributors: Dorean Nabukalu, Anthony Ndyanabo, Joseph Ssekasanvu, Hadijja Nakawooya, Jessica Nakukumba, Grace N. Kigozi, Betty S. Nantume, Nampijja Resty, Jedidah Kambasu, Margaret Nalugemwa, Regina Nakabuye, Lawrence Ssebanobe, Justine Nankinga, Adrian Kayiira, Gorreth Nanfuka, Ruth Ahimbisibwe, Stephen Tomusange, Ronald M. Galiwango, Sarah Kalibbali, Margaret Nakalanzi, Joseph Ouma Otobi, Denis Ankunda, Joseph Lister Ssembatya, John Baptist Ssemanda, Robert Kairania, Emmanuel Kato, Alice Kisakye, James Batte, James Ludigo, Abisagi Nampijja, Steven Watya, Kighoma Nehemia, Sr. Margaret Anyokot, Joshua Mwinike, George Kibumba, Paschal Ssebowa, George Mondo, Francis Wasswa, Agnes Nantongo, Rebecca Kakembo, Josephine Galiwango, Geoffrey Ssemango, Andrew D. Redd, John Santelli, Caitlin E. Kennedy, Jennifer Wagman

RCCS Ethics Approvals

The RCCS was approved by the Uganda Virus Research Institute Research and Ethics Committee, the Uganda National Council for Science and Technology, and the Western Institutional Review Board (Olympia, WA).

History of the RCCS and study design

Communities for the Rakai Community Cohort Study originally were selected as part of a community-randomized trial on treatment of sexually transmitted infections for HIV prevention. The original selection criteria were location in south and central regions of Rakai district, year-round road access, population stability, and projected HIV incidence of up to 2.0/100pys. The decision on number and size of the communities were based on providing adequate power for the original trial.[1] After the trial, these communities continued to be followed, but under the auspices of an observational cohort study which continues to this day.

RCCS recruitment and follow-up

To identify eligible cohort participants, a household census enumerates all persons by gender, age, and duration of residence, regardless of whether they are present or currently absent. After the census, the RCCS surveys all present, age-eligible residents providing written informed consent. Two attempts are made to contact individuals who are censused and eligible but who do not participate in the surveys. In recent surveys, we performed mobile phone outreach to survey participants from prior rounds who were not present at subsequent surveys. There were no specific incentives for follow-up given to the HIV incidence cohort, but all participants were compensated for time and travel.

Combination HIV Prevention Scale-Up

Male circumcision. Prior to 2002, with the exception of Muslims (~12% of the male cohort), MC services in Rakai were very limited. From 2003 to 2006, RHSP conducted a large randomized controlled trial of MC which demonstrated significant efficacy for HIV prevention in men.[2] In 2007, the World Health Organization recommended that MC be part of CHP strategies,[3] and RHSP first provided MC services to trial controls and then free MC services throughout Rakai, with funding from the U.S. President's Emergency Plan for AIDS Relief (PEPFAR).[4]

Antiretroviral therapy. Prior to 2004, ART availability in Rakai was very limited. In 2004, with PEPFAR support, RHSP provided ART to RCCS participants and non-RCCS clients through mobile and static clinics.[5] In 2013, services were provided by Ministry of Health (MOH) personnel with RHSP supervision and monitoring ("District Led Programming"). From 2004, the CD4 cell count criteria for ART initiation was <250 cells/mm3, subsequently raised to <350 cells/mm3 in 2011 and to <500 cells/mm3 in January 2014 along with ART provision to pregnant and breastfeeding women, serodiscordant couples, and key populations (e.g., commercial sex workers, truckers, and fisherfolk) regardless of CD4 count. HIV-positive individuals on ART were monitored clinically, with six monthly CD4+ cell count and viral load testing, the results of which were routinely provided to patients as part of adherence counseling. ART was also available through non-governmental organizations.

HIV testing services, condoms, and behavioral interventions. Free HIV testing services were provided to all RCCS participants. Condoms and behavioral counseling were also provided and HIV prevention messaging campaigns were implemented.[6] Intervention studies have also been conducted by RHSP to improve linkage and adherence to pre-ART and ART care, leading to the integration of peer supporters and “treatment buddies” into routine care.[5,7,8]

RCCS Laboratory Methods

All RCCS participants are offered free voluntary counseling and HIV testing. The vast majority (>90%) of participants over the lifetime of the cohort consent to be tested and to receive their results. A small percentage (<5%) agree to be tested but choose not to receive their HIV results. Since 2011, HIV rapid tests results are returned to participants immediately through on-site post-test counselors. Prior to rapid testing, a community-based counselor system was used to return results to participants.

As HIV testing methods evolved, RCCS testing algorithms have changed accordingly (see next section for more details). In brief, prior to October 2011, HIV testing used enzyme immunoassays (EIAs) with confirmation via Western Blot. Subsequently a field-validated parallel three test rapid HIV testing algorithm was introduced with demonstrated high sensitivity (>99.5%) and specificity (>99.5%).[9,10] All rapid test positives are confirmed by two EIAs, with Western blot or PCR for discordant EIA results.

HIV viral loads were retrospectively measured for this analysis using stored sera for all HIV-positive participants in surveys 8 (Jul.2009) and 12 (Jan.2016). HIV-1 viral load testing was performed using the Abbott RealTime assay (Abbott Molecular, Inc., Des Plaines, IL 60018).

References

1. Wawer MJ, Sewankambo NK, Serwadda D, et al. Control of sexually transmitted diseases for AIDS prevention in Uganda: a randomised community trial. Rakai Project Study Group. Lancet 1999;353:525-35.
2. Gray RH, Kigozi G, Serwadda D, et al. Male circumcision for HIV prevention in men in Rakai, Uganda: a randomised trial. Lancet 2007;369:657-66.
3. WHO/UNAIDS Technical Consultation on Male Circumcision and HIV Prevention: Research Implications for Policy and Programming (2007). New Data on Male Circumcision and HIV Prevention: Policy and Programme Implications. .
4. Government of Uganda, Ministry of Health. Safe Male Circumcision Policy. January 2010.
5. Chang LW, Kagaayi J, Nakigozi G, et al. Effect of peer health workers on AIDS care in Rakai, Uganda: a cluster-randomized trial. PloS one 2010;5:e10923.
6. Murphy EM, Greene ME, Mihailovic A, Olupot-Olupot P. Was the “ABC” Approach (Abstinence, Being Faithful, Using Condoms) Responsible for Uganda's Decline in HIV? PLoS medicine 2006;3:e379.
7. Chang LW, Nakigozi G, Billioux VG, et al. Effectiveness of peer support on care engagement and preventive care intervention utilization among pre-antiretroviral therapy, HIV-infected adults in Rakai, Uganda: a randomized trial. AIDS Behav 2015;19:1742-51.
8. Nakigozi G, Makumbi FE, Bwanika JB, et al. Impact of Patient-Selected Care Buddies on Adherence to HIV Care, Disease Progression, and Conduct of Daily Life Among Pre-antiretroviral HIV-Infected Patients in Rakai, Uganda: A Randomized Controlled Trial. J Acquir Immune Defic Syndr 2015;70:75-82.
9. Gray RH, Makumbi F, Serwadda D, et al. Limitations of rapid HIV-1 tests during screening for trials in Uganda: diagnostic test accuracy study. Bmj 2007;335:188.
10. Kagulire SC, Opendi P, Stamper PD, et al. Field evaluation of five rapid diagnostic tests for screening of HIV-1 infections in rural Rakai, Uganda. International journal of STD & AIDS 2011;22:308-

History of HIV Testing Algorithms in the RCCS

The RCCS HIV testing algorithm has changed over time. Details of testing methods are described below.

EIA=Enzyme immunoassay; WB=Western blot; PCR= Polymerase Chain Reaction test

EIA testing kits	Time Introduced
Organon Teknika<br>Cambridge	1999
Cambridge<br>Vironostika HIV Uni-form 11 plus O	2003
Vironstika Uni-form 11 plus O<br>Welcozyme	2004
Murex HIV 1.2<br>Vironostika HIV Uni-form 11 plus O<br>BioRad GS HIV -1/HIV-2 plus O	Nov 2004
Vironostika Ag/Ab<br>Murex HIV 1.2	2013
BioRad GS HIV -1/HIV-2 plus O<br>Vironstika HIV Ag/Ab	2014
Murex Ag/Ab<br>BioRad Ag/Ab	Dec 2015
Bioelisa HIV-1+2 Ag/Ab<br>Murex Ag/Ab	July 2016

1. ***Prior to 1st April 2009 (EIA testing in parallel)***

   Two EIAs were run in parallel (see above table for list of all EIA tests performed). When EIA results were discordant a WB was performed.

2. ***1st April 2009 (Serial EIA testing)***

   EIAs were run in series and participants who had already tested HIV seropositive twice were no longer tested.

   Samples testing seronegative on Murex HIV 1.2. were reported as HIV-seronegative and no additional testing was performed.

   Samples from persons testing HIV-seropositive for the first time with Murex 1.2 were also run with the Vironostika HIV Uni-form 11 plus O.

   Concordant HIV seropositive results were reported as seropositive and discordant results were further evaluated on WB.

   Indeterminate WB's were recorded as such (uncertain) and an RNA PCR (Roche Amplicor 1.5) was performed.

3. ***October 2011 (Field validation or rapid HIV tests with EIA)***

   Rapid HIV testing was introduced. Determine and Stat Pak rapid tests were run in parallel. In the case of rapid discordant results, samples were further tested on Unigold according to Ugandan National HIV guidelines.

   In order to assess the validity of rapid test results (obtained in the community), all rapid seropositive samples, rapid discordant samples as well as weak positive rapid samples were run on two EIAs starting with Murex and then proceeding to Vironostika HIV Uni-form 11 plus O when the Murex test result was seropositive. If the sample was seronegative on Vironostika, the sample was further tested with BioRad (GS HIV-1/2 plus O (Bio Rad)). For those samples that were seropositive on BioRad, a WB or RNA PCR was also performed.

4. ***January 2012 (Rapid HIV testing algorithm with confirmatory EIA testing for rapid seropositive and discordant results)***

   The rapid test algorithm was adopted in lieu of serial EIA tests following the rapid test field validation (see above). All new seropositives were confirmed by two EIAs, with WB or RNA PCR for discordant EIAs.

5. ***January 2013***
   Tertiary EIA and WB testing was discontinued after systematic comparison retaining and excluding results. Two EIAs were used to confirm seropositive rapid results and to resolve rapid discordant results. If EIAs were discordant, WB or RNA PCR were performed.

6. ***December 2015-present***
   Murex was used for first-line confirmatory testing for all rapid HIV seropositive and discordant test results. BioRad was used for second-line confirmatory testing.

Summary and description of variables included in primary multivariate regression analyses of HIV incidence

Variables included in multivariate model	Description of variable	Multivariate regression models				
		***Model I**: All participants (Table 2A, Supplemental Table 3A)*	***Model II:** Women only (Table 2B, Supplemental Table 3B)*	***Model III:** Men only (Table 2C; Supplemental Table 3C)*	***Model IV**: Uncircumcised men only (Table 2D: Supplemental Table 3D)*	***Model V:** Circumcised men only (Supplemental Table 3E)*
Survey/Calendar time (primary exposure variable)	References the seven RCCS survey periods (calendar time) during which HIV incidence was assessed. The reference period includes visit intervals prior to CHP scale-up which began with the sixth survey. Incidence is reported at the visit incidence cases were first detected. The period-specific mean incidence rates during the survey interval between present and prior survey were estimated.	x	x	x	x	x
Age (years)	Individual-level time-varying age was analyzed as a categorical variable (e.g., five-year age groups with exception of the oldest age group, 40-49 years). Age was recorded by interviewer using the participant verification form prior to the RCCS survey.	x	x	x	x	x
Gender I.	Individual-level time-varying categorical variable collected at time of participant verification and survey. Included the following groups: 1) Women (reference) 2) uncircumcised men and 3) circumcised men	x				

Gender II.	Categorical variable assessed by interviewer at time of participant verification. Included the following categories 1) women (reference) 2) men (irrespective of male circumcision status)					
Education	Time-fixed variable collected at baseline visit. Level listed is highest educational level reached but not necessarily achieved. Categories included none, primary, secondary, and university/technical education-levels. Data were self-reported.	x	x	x	x	x
Male circumcision status	Individual-level time-varying variable collected at baseline and follow-up visits from men. Data were self-reported.			x		
Number of sex partners in the last year	Individual-level time-varying variable collected at baseline and follow-up visits. Analyzed as a categorical variable (None, 1, 2, 3 or more). Data were self-reported.	x	x	x	x	x
Sex with partners outside the community in the last year	Individual-level time-varying variable collected at baseline and follow-up visits. Data were self-reported.	x	x	x	x	x
Self-reported genital ulcer disease in the last year	Individual-level time-varying variable collected at baseline and follow-up visits. Data were self-reported.	x	x	x	x	x
Self-report of non-marital partnerships and consistent condom use	Individual-level time-varying variable collected at baseline and follow-up visits. Data were self-reported.	x	x	x	x	x
Birth Cohort	Individual-level time-fixed covariate; Categorical variable based on 5-year birth cohort using birth year beginning with individuals born in 1960 (e.g. 1960-1964; 1965-1969, etc.); Individuals born prior to 1960 were analyzed as a single birth cohort.					

Community type	Community-level time-varying variable. Individuals were classified as living in agrarian or trading communities as previously described. *	x	x	x	x	x
Community HIV prevalence*	Community-level time varying variable. Defined as mean HIV prevalence in an individual's community at time of survey and analyzed as a categorical variable with ranges based on distribution of all community HIV prevalence estimates.	x	x	x	x	x

*Chang et al. Heterogeneity of the HIV epidemic in agrarian, trading, and fishing communities in Rakai, Uganda: an observational epidemiological study. *The Lancet HIV*. 2016. 3 (8), e388-e396; **Total community HIV prevalence (rather than HIV prevalence in the opposite gender) was used for gender stratified analyses because HIV incidence in one gender is both directly impacted by HIV prevalence in the other gender and indirectly impacted by its own prevalence

Summary and description of variables included in sensitivity analyses

Variables included in multivariate model	Description of variable	***Model VII:*** *Excluding individuals who were not sexually active in the last year (Supplemental Table 5)*	***Model VIII****: Birth Cohort model (Supplemental Table 6)*	***Construction of survey participation weights***	***Construction of censoring weights***	***Model IX****: Sensitivity analysis with stabilized inverse probability weights (Supplemental Table 7)*
Survey/Calendar time (primary exposure variable)	References the seven RCCS survey periods (calendar time) during which HIV incidence was assessed. The reference period includes visit intervals prior to CHP scale-up which began with the sixth survey. Incidence is reported at the visit cases were first detected but is in reference to the mean incidence during the survey period between present and prior survey.	x	x		x	x
Age (years)	Individual-level time-varying variable analyzed as a categorical variable (five-year age groups with exception of the oldest age group, 40-49 years). Data collected using participant verification form by interviewer prior to the RCCS survey.	x	x	x	x	x
Gender I.	Individual-level time-varying categorical variable collected at time of participant verification and survey. Included the following groups: 1) Women (reference) 2) uncircumcised men and 3) circumcised men	x	x		x	x

Gender II.	Categorical variable assessed at time of participant verification by interviewer. Included the following categories 1) women (reference) 2) men (irrespective of male circumcision status)			x		
Education	Time-fixed variable collected at baseline visit. Level listed is highest educational level reached but not necessarily achieved. Categories included none, primary, secondary, and university/technical education-levels. Data were self-reported.	x	x		x	x
Male circumcision status	Individual-level time-varying variable collected at baseline and follow-up visits from men. Data were self-reported.					
Number of sex partners in the last year	Individual-level time-varying variable collected at baseline and follow-up visits. Analyzed as a categorical variable (None, 1, 2, 3 or more). Data were self-reported.	x	x		x	x
Sex with partners outside the community in the last year	Individual-level time-varying variable collected at baseline and follow-up visits. Data were self-reported.	x	x		x	x
Self-reported genital ulcer disease in the last year	Individual-level time-varying variable collected at baseline and follow-up visits. Data were self-reported.	x	x		x	x
Self-report of non-marital partnerships and consistent condom use	Individual-level time-varying variable collected at baseline and follow-up visits. Data were self-reported.	x	x		x	x
Birth Cohort	Individual-level time-fixed covariate; Categorical variable based on 5-year birth cohort using birth year beginning with individuals born in 1960 (e.g. 1960-1964; 1965-1969, etc.); Individuals born prior to 1960			x		

	were analyzed as a single birth cohort.					
Community type	Community-level time-varying variable. Individuals were classified as living in agrarion or trading communities as previously described.*	x	x	x	x	x
Community HIV prevalence	Community-level time varying variable. Defined as mean HIV prevalence in an individual's community at time of survey and analyzed as a categorical variable with ranges based on distribution of all community HIV prevalence estimates.	x	x		x	x

*Chang et al. Heterogeneity of the HIV epidemic in agrarian, trading, and fishing communities in Rakai, Uganda: an observational epidemiological study. The Lancet HIV. 2016. 3 (8), e388-e396

Supplemental statistical methods

All statistical analyses were conducted using the R statistical software (version 3.3.2).

Analysis of HIV incidence community ART coverage, community prevalence of viremia, and community MC coverage

Scale up of ART and male circumcision coverage as well as prevalence of viremia at the community-level was assessed over the study period. ART coverage in community *i* at visit *j* was estimated as the proportion of HIV positive persons in community *i* at time *j* who self-reported ART use. Male circumcision coverage was similarly defined using self-reported data from men. The prevalence of population viremia in an RCCS community at a given visit was calculated as follows:

$$Viremia_{ij} = HIV\ prevalence_{ij} - 0.90 * ART\ coverage_{ij}$$

We multiply ART coverage by 0.90 because ~90% of self-reported ART users were suppressed at the two time points we assessed population viremia and in a prior validation study of self-reported ART use. Given that there is underreporting of ART use in the RCCS, our estimates of community-level viremia are likely an upper bound.

Next, we assessed HIV incidence and individual HIV risk as a function of community-level ART coverage which was analyzed as a categorical variable (0-10% coverage, 10-20%, 20-30%, 40-50%, >50%). Similar analyses were also performed for MC coverage (0-10% coverage, 10-20%, 20-30%, 40-50%, >50%) and prevalence of viremia (0-7.5%,7.5-10%, 10-12.5%, 12.5%-15%, 15-17.5%, >17.5%). Analyses were performed for women, men and uncircumcised men where coverage of ART and viremia were measured in the opposite sex.

Estimation stabilized inverse probability weights methods

Inverse probability weighting was implemented using methods previously described by Cole et al. (see Cole S. and Hernan M.A. *Constructing inverse probability weights for marginal structural models*. Am J Epidemiol. 2008 Sep 15; 168 (6): 656-664 for more detail). Briefly, models for survey participation and censoring were specified separately. Each person-visit was treated as an observation in the models which included the time-fixed and time-varying variables specified in the table above. Our model of survey participation included variables collected at time of census (age, gender and community of residence) whereas the censoring model included demographic and sexual behavioral variables collected at census and time of survey. The denominator of the weights was a logistic regression model for the probability of contributing person-time to the incidence cohort which was defined as the joint probability of participating in the survey and of being observed at the following survey. The numerator was the joint probability of the marginal distributions for participation and censoring. Weighted incidence rate ratios were estimated using Poisson regression assuming independence between individual participant observations conditional on observed covariates.

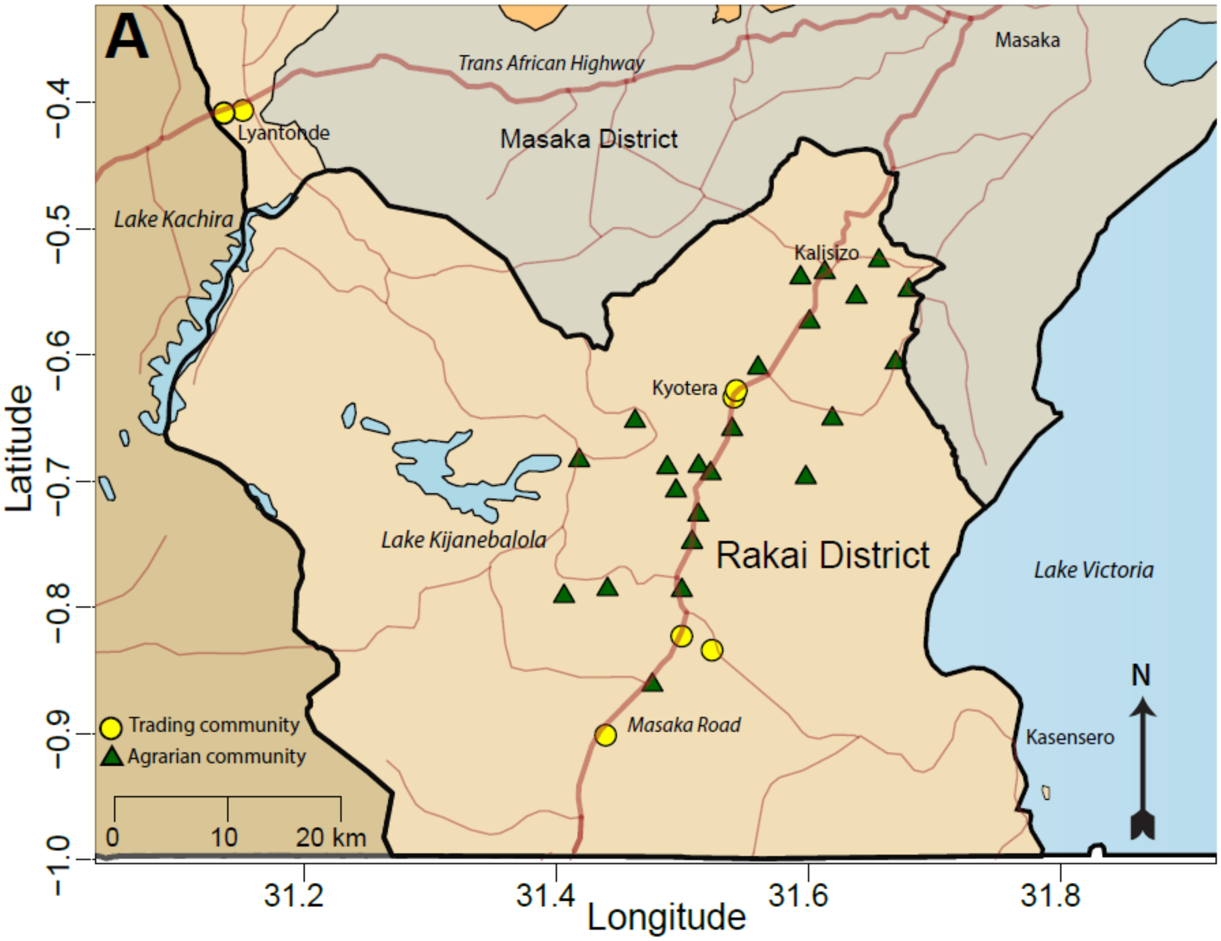

Supplemental Figure 1. Map of Rakai region and 30 continuously surveyed RCCS study communities. Agrarian communities are shown as green triangles (n=23) and trading communities (n=7) as yellow circles.

Supplemental Table 1a. Summary of RCCS participation by survey round**.**

Survey	*Participated*	*Refused**	*Away for work or school***	*Other*	*No information*
1	60.7% (5992)	20.5% (2022)	17.7% (1744)	0.8% (77)	0.3% (34)
2	64.4% (6732)	16.0% (1670)	18.0% (1881)	0.9% (96)	0.7% (69)
3	64.9% (7340)	14.8% (1677)	18.9% (2140)	0.8% (93)	0.6% (66)
4	60.0% (6856)	13.9% (1586)	24.8% (2833)	0.9% (101)	0.5% (60)
5	59.3% (7038)	10.0% (1184)	28.9% (3424)	1.2% (148)	0.6% (66)
6	64.6% (8097)	5.5% (695)	27.1% (3391)	2.5% (319)	0.2% (26)
7	63.4% (8645)	2.5% (343)	33.0% (4506)	0.9% (121)	0.2% (21)
8	65.4% (8691)	1.5% (201)	32.2% (4284)	0.7% (96)	0.2% (21)
9	65.9% (9643)	1.1% (163)	32.0% (4680)	0.8% (124)	0.1% (19)
10	66.1% (10588)	0.8% (122)	32.2% (5161)	0.7% (108)	0.2% (28)
11	65.1% (11379)	0.3% (60)	33.8% (5911)	0.5% (95)	0.2% (32)
12	66.5% (12012)	0.5% (91)	31.9% (5757)	0.5% (94)	0.6% (111)

*Includes individuals who refused to provide a blood sample for HIV serology but who responded to the questionnaire. **These individuals were not present in the community at time of survey. Two attempts were made to locate these participants.

Supplemental Table 1b. Summary of ***female*** RCCS participation by survey round.

Survey	*Participated*	*Refused**	*Away for work or school***	*Other*	*No information*
1	66.5% (3474)	20.7% (1082)	11.8% (618)	0.7% (34)	0.3% (16)
2	70.4% (3880)	15.5% (855)	12.7% (701)	0.8% (42)	0.6% (34)
3	70.7% (4189)	13.7% (813)	14.4% (852)	0.7% (40)	0.5% (32)
4	67.1% (3993)	12.9% (765)	18.7% (1112)	0.8% (45)	0.5% (32)
5	66.1% (4114)	10.1% (626)	22.0% (1369)	1.3% (81)	0.6% (37)
6	71.2% (4681)	5.5% (359)	20.8% (1365)	2.4% (156)	0.2% (14)
7	70.0% (4993)	2.5% (176)	26.6% (1894)	0.8% (59)	0.2% (11)
8	70.9% (4961)	1.3% (93)	26.9% (1885)	0.7% (50)	0.1% (9)
9	71.0% (5418)	1.0% (75)	27.2% (2074)	0.8% (60)	0.1% (9)
10	70.7% (5842)	0.6% (52)	27.8% (2300)	0.7% (58)	0.2% (16)
11	70.0% (6333)	0.3% (27)	29.1% (2629)	0.5% (45)	0.2% (15)
12	70.7% (6650)	0.4% (38)	27.6% (2599)	0.5% (47)	0.7% (69)

*Includes individuals who refused to provide a blood sample for HIV serology but who responded to the questionnaire. **These individuals were not present in the community at time of survey. Two attempts were made to locate these participants.

Supplemental Table 1c. Summary of ***male*** RCCS participation by survey round.

Survey	*Participated*	*Refused**	*Away for work or school***	*Other*	*No information*
1	54.2% (2518)	20.2% (940)	24.2% (1126)	0.9% (43)	0.4% (18)
2	57.8% (2852)	16.5% (815)	23.9% (1180)	1.1% (54)	0.7% (35)
3	58.5% (3151)	16.0% (864)	23.9% (1288)	1.0% (53)	0.6% (34)
4	52.2% (2863)	15.0% (821)	31.4% (1721)	1.0% (56)	0.5% (28)
5	51.9% (2924)	9.9% (558)	36.5% (2055)	1.2% (67)	0.5% (29)
6	57.4% (3416)	5.6% (336)	34.0% (2026)	2.7% (163)	0.2% (12)
7	56.2% (3652)	2.6% (167)	40.2% (2612)	1.0% (62)	0.2% (10)
8	59.3% (3730)	1.7% (108)	38.1% (2399)	0.7% (46)	0.2% (12)
9	60.4% (4225)	1.3% (88)	37.3% (2606)	0.9% (64)	0.1% (10)
10	61.3% (4746)	0.9% (70)	37.0% (2861)	0.6% (50)	0.2% (12)
11	59.9% (5046)	0.4% (33)	38.9% (3282)	0.6% (50)	0.2% (17)
12	61.9% (5362)	0.6% (53)	36.5% (3158)	0.5% (47)	0.5% (42)

*Includes individuals who refused to provide a blood sample for HIV serology but who responded to the questionnaire. **These individuals were not present in the community at time of survey. Two attempts were made to locate these participants.

Supplemental Table 2a. Summary of reasons for loss to follow-up in the RCCS among HIV-negative persons between the previous and listed survey visit.

Survey	*Out-migrated**	*Away***	*Refused*	*Age ineligible*	*Other reason*	*Unknown*
2	42.1% (546)	32.2% (417)	21.7% (281)	0.1% (1)	2.5% (32)	1.5% (20)
3	55.9% (1677)	24.5% (736)	16.2% (485)	0.7% (21)	1.8% (54)	0.9% (28)
4	55.1% (2167)	25.9% (1017)	12.1% (477)	5.0% (198)	1.3% (50)	0.6% (25)
5	55.2% (2206)	28.1% (1123)	8.6% (344)	5.9% (235)	1.3% (52)	0.8% (33)
6	61.7% (2159)	27.6% (965)	3.9% (138)	4.9% (170)	1.7% (58)	0.3% (12)
7	59.2% (2585)	31.8% (1388)	1.6% (70)	5.3% (232)	2.1% (90)	0.1% (3)
8	60.6% (2952)	31.5% (1532)	1.0% (50)	5.1% (246)	1.8% (87)	0.1% (4)
9	61.0% (2894)	30.9% (1466)	0.6% (27)	5.8% (273)	1.7% (80)	0.0% (2)
10	60.2% (3032)	32.6% (1641)	0.4% (21)	5.4% (273)	1.3% (66)	0.1% (3)
11	62.7% (3875)	31.5% (1947)	0.2% (11)	4.6% (282)	1.0% (63)	0.0% (2)
12	62.6% (4017)	31.4% (2014)	0.3% (21)	4.4% (281)	1.2% (76)	0.1% (9)

* Individual moved to a community not under RCCS surveillance. **Includes individuals who were away for work or school at time of survey and who could not be located after two attempts.

Supplemental Table 2b. Summary of reasons for loss to follow-up in the RCCS among ***female*** HIV-negative persons between the previous and listed survey visit.

Survey	*Out-migrated**	*Away***	*Refused*	*Age ineligible*	*Other reason*	*Unknown*
2	49.3% (333)	25.6% (173)	21.6% (146)	0.0% (0)	1.5% (10)	1.9% (13)
3	61.1% (978)	21.2% (339)	15.0% (240)	0.5% (8)	1.1% (17)	1.1% (18)
4	60.7% (1249)	21.2% (436)	10.6% (218)	5.9% (121)	1.0% (20)	0.6% (13)
5	61.2% (1261)	21.8% (449)	7.8% (161)	7.1% (146)	1.3% (27)	0.8% (17)
6	66.3% (1220)	22.3% (410)	4.2% (78)	5.5% (101)	1.3% (24)	0.3% (6)
7	64.2% (1498)	25.7% (600)	1.9% (44)	6.3% (146)	1.8% (43)	0.1% (2)
8	64.7% (1748)	26.2% (707)	1.2% (33)	6.0% (161)	1.9% (51)	0.1% (2)
9	64.5% (1724)	26.7% (714)	0.6% (17)	6.6% (176)	1.5% (39)	0.0% (1)
10	63.9% (1781)	28.6% (798)	0.4% (12)	6.0% (167)	1.0% (29)	0.0% (1)
11	66.1% (2158)	28.0% (913)	0.1% (4)	5.0% (163)	0.8% (25)	0.0% (1)
12	65.8% (2213)	28.4% (955)	0.2% (8)	4.5% (152)	0.9% (29)	0.1% (5)

* Individual moved to a community not under RCCS surveillance. **Includes individuals who were away for work or school at time of survey and who could not be located after two attempts.

Supplemental Table 2c. Summary of reasons for loss to follow-up in the RCCS among ***male*** HIV-negative persons between the previous and listed survey visit.

Survey	*Out-migrated**	*Away***	*Refused*	*Age ineligible*	*Other reason*	*Unknown*
2	34.2% (213)	39.2% (244)	21.7% (135)	0.2% (1)	3.5% (22)	1.1% (7)
3	49.9% (699)	28.3% (397)	17.5% (245)	0.9% (13)	2.6% (37)	0.7% (10)
4	48.9% (918)	31.0% (581)	13.8% (259)	4.1% (77)	1.6% (30)	0.6% (12)
5	48.9% (945)	34.9% (674)	9.5% (183)	4.6% (89)	1.3% (25)	0.8% (16)
6	56.5% (939)	33.4% (555)	3.6% (60)	4.1% (69)	2.0% (34)	0.4% (6)
7	53.4% (1087)	38.7% (788)	1.3% (26)	4.2% (86)	2.3% (47)	0.0% (1)
8	55.5% (1204)	38.0% (825)	0.8% (17)	3.9% (85)	1.7% (36)	0.1% (2)
9	56.5% (1170)	36.3% (752)	0.5% (10)	4.7% (97)	2.0% (41)	0.0% (1)
10	55.6% (1251)	37.5% (843)	0.4% (9)	4.7% (106)	1.6% (37)	0.1% (2)
11	58.9% (1717)	35.5% (1034)	0.2% (7)	4.1% (119)	1.3% (38)	0.0% (1)
12	59.0% (1804)	34.7% (1059)	0.4% (13)	4.2% (129)	1.5% (47)	0.1% (4)

* Individual moved to a community not under RCCS surveillance. **Includes individuals who were away for work or school at time of survey and who could not be located after two attempts.

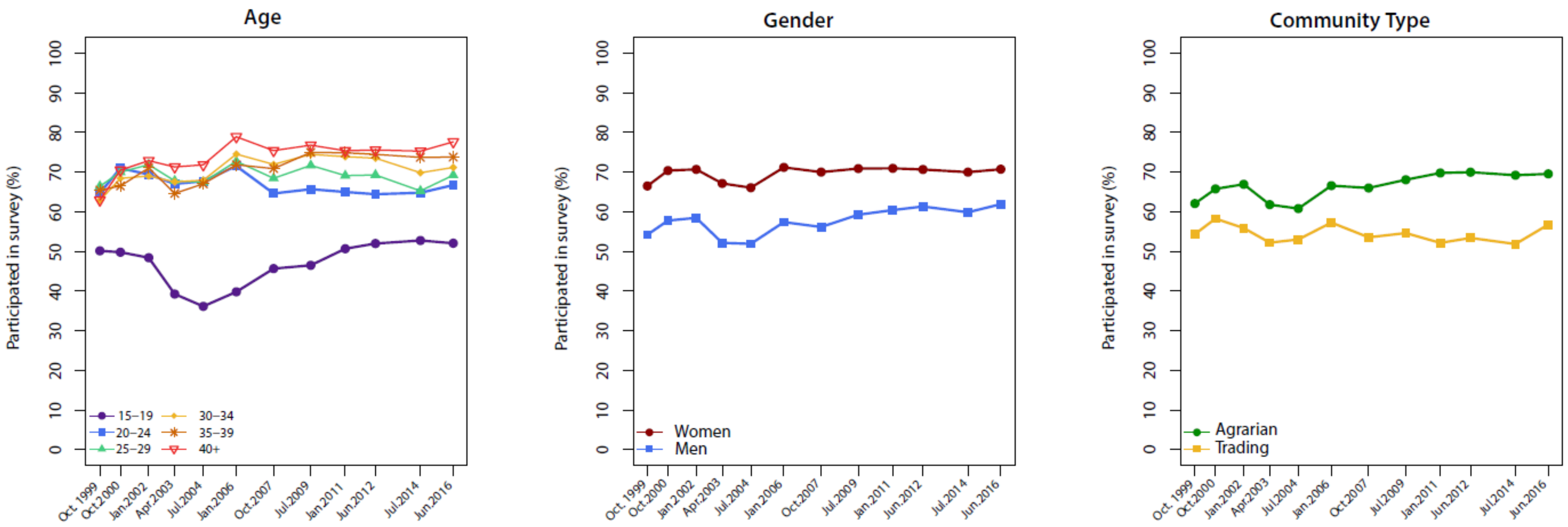

Supplemental Figure 2. Summary of RCCS survey participation over calendar time by participant age, gender, and their community-type of residence. Participation was lowest among the very young, men, and persons residing in trading communities.

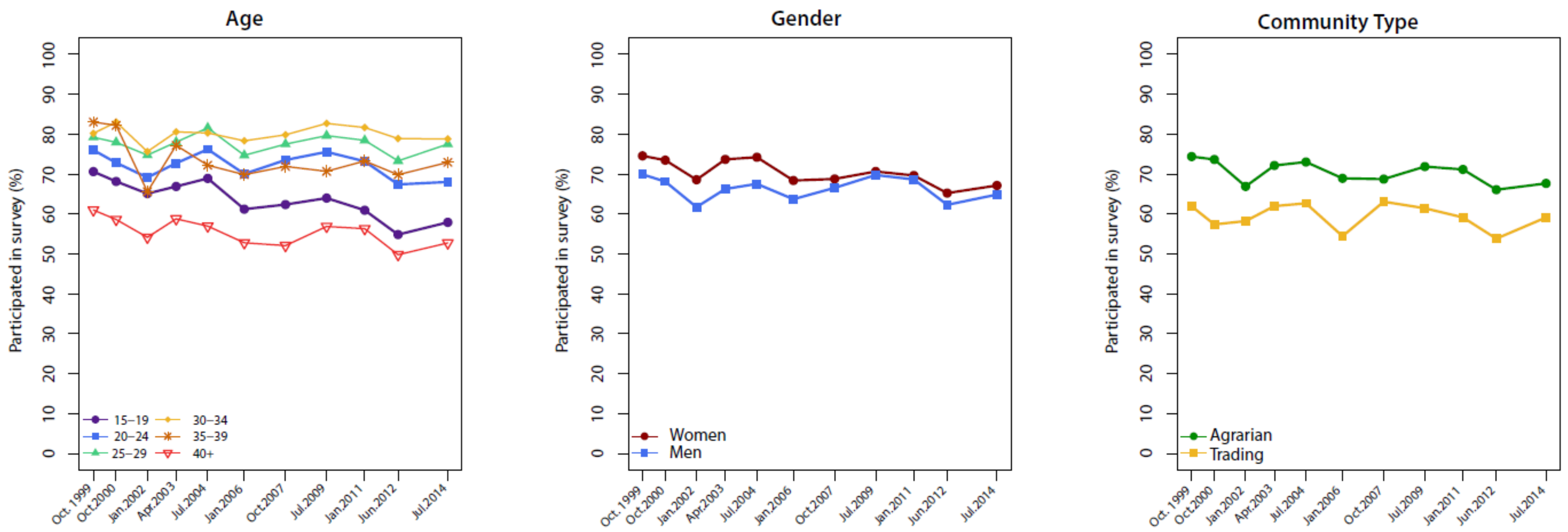

Supplemental Figure 3. Summary of loss to follow-up (after a single survey interval) among HIV-negative RCCS participants over calendar time by participant age, gender, and their community-type of residence.

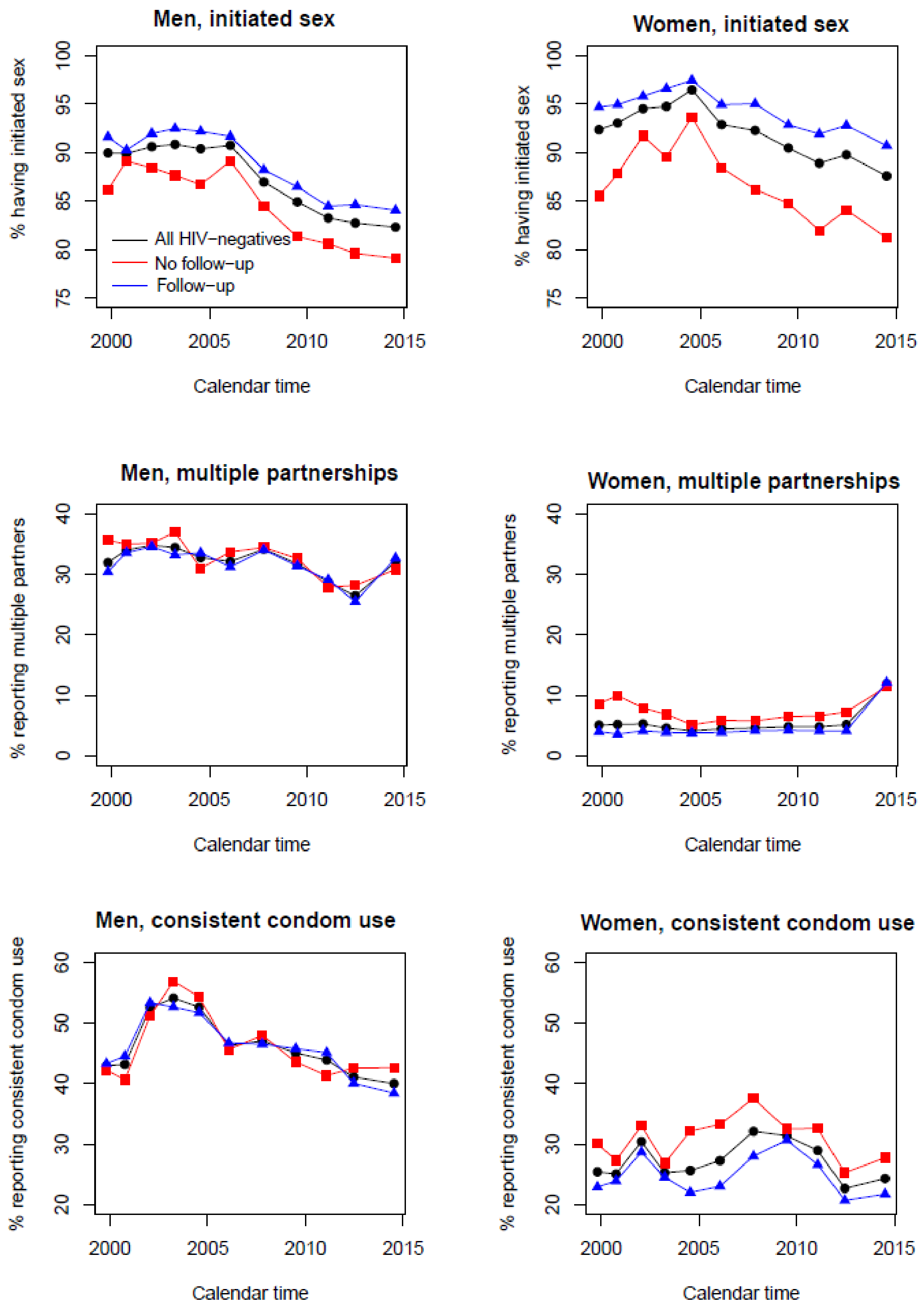

Supplemental Figure 4: Sexual behaviors among HIV-negative participants who were interviewed at the following survey and those who were not by calendar time. Condom use was assessed among those reporting casual/non-stable sexual partnerships only because condom use within marital unions is low.

Supplemental Table 3A. Probability of self-reported ART use by survey period and gender with and without inverse probability weights for survey participation.

	**ARV coverage estimates by survey**		
Survey	*Women and Men*	*Women*	*Men*
6	11.8% (9.8%,14.0%)	13.7% (11.0%,16.7%)	8.1% (11%,16.7%)
7	17.5% (15.1%,20.0%)	18.2% (15.3%,21.5%)	15.8% (15.3%,21.5%)
8	22.5% (19.9%,25.4%)	23.8% (20.5%,27.5%)	20.0% (20.5%,27.5%)
9	26.8% (24.1%,29.7%)	29.4% (26.0%,33.2%)	21.6% (26.0%,33.2%)
10	35.6% (32.6%,38.7%)	38.7% (34.9%,42.8%)	29.5% (34.9%,42.8%)
11	54.7% (51.0%,58.5%)	58.7% (54.1%,63.5%)	46.3% (54.1%,63.5%)
12	68.9% (64.8%,73.2%)	72.3% (67.3%,77.5%)	61.3% (67.3%,77.5%)

	**ARV coverage estimates with inverse probability weights by survey**		
Survey	*Women and Men*	*Women*	*Men*
6	11.3% (9.6%,13.2%)	13.2% (10.9%,15.8%)	8.1% (5.9%,10.8%)
7	16.6% (14.7%,18.7%)	17.4% (14.9%,20.1%)	15.3% (12.3%,18.8%)
8	21.6% (19.4%,24.0%)	23.0% (20.2%,26.1%)	19.3% (16.0%,23.1%)
9	25.7% (23.5%,28.1%)	28.6% (25.6%,31.8%)	20.8% (17.5%,24.4%)
10	35.0% (32.5%,37.7%)	38.1% (34.9%,41.6%)	29.6% (25.8%,33.7%)
11	53.8% (50.7%,57.0%)	58.5% (54.5%,62.6%)	45.0% (40.3%,50.1%)
12	68.1% (64.7%,71.8%)	71.5% (67.2%,76.1%)	61.0% (55.3%,67.2%)

Supplemental Table 3B. Probability of self-reported male circumcision (MC) status among men by survey period and HIV status with and without inverse probability weights for survey participation

	**MC coverage estimates by survey**		
Survey	*All men*	*HIV-negative men*	*HIV-positive men*
1	15.0% (13.6%,16.6%)	15.8% (14.2%,17.5%)	9.1% (5.9%,13.2%)
2	14.8% (13.4%,16.2%)	15.4% (13.9%,16.9%)	9.5% (6.4%,13.6%)
3	16.5% (15.1%,18.0%)	17.0% (15.6%,18.6%)	11.9% (8.6%,16.1%)
4	17.4% (15.9%,19.0%)	18.0% (16.4%,19.7%)	11.7% (8.1%,16.3%)
5	18.6% (17.1%,20.2%)	19.0% (17.4%,20.8%)	15.2% (11.3%,20.0%)
6	22.2% (20.6%,23.8%)	22.6% (20.9%,24.3%)	18.5% (14.2%,23.5%)
7	28.3% (26.6%,30.1%)	28.3% (26.5%,30.1%)	28.9% (23.7%,34.8%)
8	33.4% (31.5%,35.2%)	33.3% (31.4%,35.3%)	33.7% (28.3%,39.7%)
9	39.4% (37.5%,41.3%)	39.5% (37.5%,41.5%)	38.7% (33.2%,44.7%)
10	43.3% (41.5%,45.2%)	43.3% (41.4%,45.3%)	43.2% (37.7%,49.3%)
11	52.2% (50.3%,54.3%)	52.7% (50.6%,54.8%)	47.7% (41.8%,54.2%)
12	59.3% (57.3%,61.4%)	60.1% (58.0%,62.3%)	50.5% (44.3%,57.3%)
	**MC coverage estimates with inverse probability weights by survey**		
Survey	*All men*	*HIV-negative men*	*HIV-positive men*
1	15.4% (14.3%,16.6%)	16.1% (14.9%,17.3%)	9.4% (6.9%,12.5%)
2	14.9% (13.9%,16.0%)	15.5% (14.3%,16.7%)	9.4% (6.9%,12.5%)
3	16.8% (15.8%,18.0%)	17.3% (16.1%,18.5%)	12.6% (9.8%,16.0%)
4	17.8% (16.8%,19.0%)	18.4% (17.2%,19.6%)	11.8% (8.9%,15.2%)
5	19.0% (17.9%,20.1%)	19.3% (18.1%,20.5%)	15.6% (12.4%,19.3%)
6	22.4% (21.2%,23.6%)	22.7% (21.5%,24.0%)	19.0% (15.4%,23.1%)
7	28.1% (26.9%,29.4%)	28.0% (26.7%,29.4%)	29.3% (25.0%,34.0%)
8	33.3% (31.9%,34.7%)	33.2% (31.8%,34.7%)	33.6% (29.2%,38.5%)
9	39.4% (37.9%,40.8%)	39.4% (37.9%,41.0%)	38.8% (34.3%,43.7%)
10	43.2% (41.8%,44.7%)	43.3% (41.8%,44.8%)	42.8% (38.2%,47.7%)
11	52.9% (51.4%,54.4%)	53.5% (51.8%,55.1%)	46.7% (41.9%,51.9%)
12	60.2% (58.5%,61.8%)	61.0% (59.3%,62.7%)	49.8% (44.7%,55.4%)

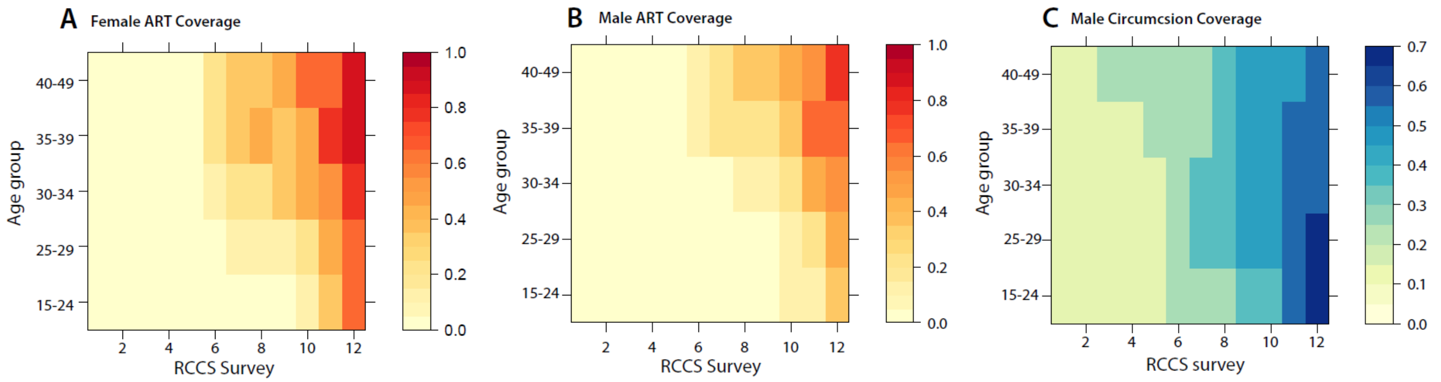

Supplementary Figure 5. ART and male circumcision coverage by age group and gender. A) ART coverage in women by 5; B) ART coverage in men; C) Male circumcision

Supplemental Table 4A. HIV incidence and unadjusted and adjusted incidence rate ratios comparing HIV incidence among ***all participants in HIV incident cohort (Model I)*** at each visit interval during combination HIV prevention (CHP) scale-up compared to HIV incidence in the period prior to scale-up.

Variable	*Incident cases*	*person-years*	*Incidence per 100 py (95%CI)*	*IRR (95%CI)*	*p-value*	*adjRR (95%CI)*	*p-value*
**Survey(s)**							
Pre-CHP (2-5)	254	21765	1.17 (1.03,1.32)	Ref.	-	Ref	-
Jan.2006 (6)	86	7773	1.11 (0.89,1.36)	0.95 (0.74,1.21)	0.659	0.94 (0.73,1.20)	0.608
Oct.2007 (7)	105	8769	1.20 (0.98,1.44)	1.02 (0.82,1.29)	0.839	1.00 (0.79,1.26)	0.999
Jul.2009 (8)	125	10201	1.23 (1.02,1.45)	1.05 (0.85,1.30)	0.670	0.95 (0.76,1.18)	0.619
Jan.2011 (9)	105	9815	1.07 (0.88,1.29)	0.91 (0.73,1.15)	0.436	0.94 (0.74,1.19)	0.602
Jun.2012 (10)	86	10352	0.83 (0.67,1.02)	0.71 (0.55,0.91)	0.006	0.72 (0.56,0.93)	0.012
Jul.2014 (11)	87	13159	0.66 (0.53,0.81)	0.56 (0.44,0.72)	<0.001	0.60 (0.47,0.78)	<0.001
Jan.2016 (12)	83	12593	0.66 (0.53,0.81)	0.56 (0.44,0.72)	<0.001	0.58 (0.45,0.76)	<0.001
**Age (years)**							
15-19	61	11218	0.54 (0.42,0.69)	0.44 (0.33,0.59)	<0.001	0.67 (0.49,0.91)	0.011
20-24	224	18233	1.23 (1.07,1.40)	Ref.	-	Ref	-
25-29	268	18964	1.41 (1.25,1.59)	1.15 (0.96,1.37)	0.126	1.07 (0.89,1.30)	0.473
30-34	178	16782	1.06 (0.91,1.22)	0.86 (0.71,1.05)	0.136	0.82 (0.66,1.02)	0.076
35-39	103	12770	0.81 (0.66,0.97)	0.65 (0.52,0.83)	<0.001	0.65 (0.50,0.83)	0.001
>39 years	97	16460	0.59 (0.48,0.71)	0.48 (0.38,0.61)	<0.001	0.46 (0.35,0.61)	<0.001
**Gender**							
Women	549	52108	1.05 (0.97,1.14)	Ref.	-	Ref	-
Uncircumcised men	283	25893	1.09 (0.97,1.23)	1.04 (0.90,1.20)	0.609	0.80 (0.66,0.97)	0.020
Circumcised men	97	16256	0.60 (0.49,0.72)	0.57 (0.46,0.70)	<0.001	0.48 (0.37,0.62)	<0.001
**Education**							
None	52	3876	1.34 (1.01,1.74)	1.25 (0.94,1.66)	0.132	1.20 (0.90,1.60)	0.209
Primary	627	58319	1.08 (0.99,1.16)	Ref.	-	Ref	-
Secondary	216	24885	0.87 (0.76,0.99)	0.81 (0.69,0.94)	0.007	0.86 (0.73,1.01)	0.059
Tertiary/Technical school	28	6565	0.43 (0.29,0.60)	0.40 (0.27,0.58)	<0.001	0.40 (0.27,0.59)	<0.001
**Number of sex partners in the last year**							

None	57	13799	0.41 (0.31,0.53)	0.45 (0.34,0.59)	<0.001	0.44 (0.32,0.62)	<0.001
One	573	62408	0.92 (0.85,1.00)	Ref.	-	Ref	-
Two	189	12368	1.53 (1.32,1.76)	1.67 (1.41,1.97)	<0.001	1.58 (1.24,2.01)	<0.001
Three or more	103	4730	2.18 (1.78,2.63)	2.37 (1.92,2.93)	<0.001	1.93 (1.44,2.59)	<0.001
**Sex with partners outside of the community in last year**							
No	557	62874	0.89 (0.81,0.96)	Ref.	-	Ref	-
Yes	374	31554	1.19 (1.07,1.31)	1.34 (1.17,1.53)	<0.001	1.28 (1.07,1.54)	0.007
**Self-reported genital ulcer disease in the last year**							
No	735	85574	0.86 (0.80,0.92)	Ref.	-	Ref	-
Yes	196	8829	2.22 (1.92,2.55)	2.59 (2.21,3.03)	<0.001	2.07 (1.76,2.43)	<0.001
**Non-marital relationships and consistent condom use***							
Stable partners only	441	61373	0.72 (0.65,0.79)	Ref.	-	Ref	-
Non-stable partners, inconsistent condom use	368	21417	1.72 (1.55,1.90)	2.40 (2.09,2.75)	<0.001	1.48 (1.14,1.92)	0.003
Non-stable partners, consistent condom use	122	11638	1.05 (0.87,1.25)	1.46 (1.2,1.79)	<0.001	1.06 (0.80,1.41)	0.691
**Community type**							
Agrarian	763	79850	0.96 (0.89-1.02)	Ref.	-	Ref	-
Trading	168	14577	1.15 (0.99,1.34)	1.21 (1.02,1.43)	0.029	0.86 (0.71,1.05)	0.151
**Community HIV prevalence**							
0.4-10%	128	17887	0.72 (0.60,0.85)	Ref.	-	Ref	-
10-13%	348	38913	0.89 (0.80,0.99)	1.25 (1.02,1.53)	0.032	1.48 (1.20,1.82)	<0.001
13-15%	199	20003	0.99 (0.86,1.14)	1.39 (1.11,1.74)	0.004	1.66 (1.32,2.08)	<0.001
15-20%	184	13781	1.34 (1.15,1.54)	1.86 (1.49,2.34)	<0.001	1.98 (1.56,2.52)	<0.001
>20%	72	3844	1.87 (1.47,2.34)	2.62 (1.96,3.50)	<0.001	2.97 (2.13,4.16)	<0.001

IRR=Incidence rate ratio, adjIRR=adjusted incidence rate ratio; *Stable partners include marital unions and long-term consensual partnerships; Casual partners include all other types partnerships. Condom use only assessed among individuals reporting casual/non-stable partnerships because of very low levels (<5%) of condom use within stable partnerships. Those reporting casual/non-stable partners may also have had stable partners.

Supplemental Table 4B. HIV incidence and unadjusted and adjusted incidence rate ratios comparing HIV incidence among ***female participants in HIV incident cohort (Model II)*** at each visit interval during combination HIV prevention (CHP) scale-up compared to HIV incidence in the period prior to scale-up.

Variable	*Incident cases*	*person-years*	*Incidence per 100 py (95%CI)*	*IRR (95%CI)*	*p-value*	*adjRR (95%CI)*	*p-value*
**Survey(s)**							
Pre-CHP (2-5)	145	12409	1.17 (0.99,1.37)	Ref.	-	Ref	-
Jan.2006 (6)	50	4425	1.13 (0.84,1.47)	0.97 (0.70,1.33)	0.836	0.98 (0.71,1.35)	0.903
Oct.2007 (7)	65	4978	1.31 (1.01,1.65)	1.12 (0.83,1.50)	0.459	1.10 (0.82,1.48)	0.515
Jul.2009 (8)	67	5610	1.19 (0.93,1.50)	1.02 (0.76,1.36)	0.885	0.91 (0.68,1.23)	0.548
Jan.2011 (9)	61	5319	1.15 (0.88,1.46)	0.98 (0.73,1.32)	0.898	0.98 (0.72,1.33)	0.888
Jun.2012 (10)	50	5587	0.89 (0.67,1.17)	0.77 (0.56,1.05)	0.102	0.75 (0.54,1.05)	0.096
Jul.2014 (11)	55	7090	0.78 (0.59,1.00)	0.66 (0.49,0.90)	0.010	0.65 (0.47,0.91)	0.012
Jan.2016 (12)	56	6689	0.84 (0.64,1.08)	0.72 (0.53,0.97)	0.034	0.68 (0.50,0.94)	0.021
**Age (years)**							
15-19	48	5324	0.90 (0.67,1.18)	0.60 (0.44,0.84)	0.002	0.66 (0.45,0.95)	0.027
20-24	150	10030	1.50 (1.27,1.75)	Ref.	-	Ref	-
25-29	150	10998	1.36 (1.16,1.59)	0.91 (0.73,1.14)	0.419	0.93 (0.73,1.18)	0.533
30-34	87	9553	0.91 (0.73,1.12)	0.61 (0.47,0.79)	<0.001	0.62 (0.46,0.82)	0.001
35-39	57	6997	0.81 (0.62,1.04)	0.54 (0.40,0.74)	<0.001	0.53 (0.38,0.74)	<0.001
>39 years	57	9206	0.62 (0.47,0.79)	0.41 (0.30,0.56)	<0.001	0.38 (0.27,0.54)	<0.001
**Education**							
None	40	2555	1.57 (1.13,2.10)	1.43 (1.02,1.99)	0.036	1.47 (1.05,2.05)	0.025
Primary	345	31476	1.10 (0.98,1.22)	Ref.	-	Ref	-
Secondary	149	14368	1.04 (0.88,1.21)	0.95 (0.78,1.15)	0.576	0.90 (0.73,1.10)	0.299
Tertiary/Technical school	11	3435	0.32 (0.17,0.55)	0.29 (0.16,0.53)	<0.001	0.30 (0.16,0.55)	<0.001
**Number of sex partners in the last year**							
None	44	7416	0.59 (0.43,0.79)	0.58 (0.43,0.80)	0.001	0.45 (0.30,0.69)	<0.001
One	428	42127	1.02 (0.92,1.12)	Ref.	-	Ref	-
Two	56	1815	3.09 (2.35,3.97)	3.04 (2.30,4.01)	<0.001	1.80 (1.27,2.55)	0.001
Three or more	14	162	8.63 (4.86,13.96)	8.50 (4.98,14.51)	<0.001	3.42 (1.98,5.9)	<0.001

**Sex with partners outside of the community in last year**								
No	347	38314	0.91 (0.81,1.00)	Ref.	-	Ref	-
Yes	202	13794	1.46 (1.27,1.68)	1.62 (1.36,1.93)	<0.001	1.42 (1.1,1.83)	0.008
**Self-reported genital ulcer disease in the last year**								
No	432	46540	0.93 (0.84,1.02)	Ref.	-	Ref	-
Yes	117	5561	2.10 (1.75,2.51)	2.27 (1.85,2.78)	<0.001	2.02 (1.64,2.49)	<0.001
**Non-marital relationships and consistent condom use***								
Stable partners only	300	39408	0.76 (0.68,0.85)	Ref.	-	Ref	-
Non-stable partners, inconsistent condom use	198	9499	2.08 (1.81,2.39)	2.74 (2.29,3.28)	<0.001	1.43 (0.95,2.14)	0.088
Non-stable partners, consistent condom use	51	3201	1.59 (1.20,2.07)	2.09 (1.55,2.82)	<0.001	1.14 (0.72,1.78)	0.575
**Community type**								
Agrarian	440	43464	1.01 (0.92,1.11)	Ref.	-	Ref	-
Trading	109	8644	1.26 (1.04,1.51)	1.25 (1.01,1.54)	0.043	0.95 (0.74,1.21)	0.677
**Community HIV prevalence**								
0.4-10%	77	9823	0.78 (0.62,0.97)	Ref.	-	Ref	-
10-13%	210	21303	0.99 (0.86,1.13)	1.26 (0.97,1.63)	0.085	1.39 (1.06,1.81)	0.017
13-15%	112	10945	1.02 (0.85,1.22)	1.31 (0.98,1.75)	0.073	1.47 (1.09,1.99)	0.011
15-20%	108	7821	1.38 (1.14,1.66)	1.76 (1.31,2.36)	<0.001	1.74 (1.27,2.38)	<0.001
>20%	42	2215	1.90 (1.38,2.53)	2.42 (1.65,3.54)	<0.001	2.38 (1.55,3.67)	<0.001

IRR=Incidence rate ratio, adjIRR=adjusted incidence rate ratio; *Stable partners include marital unions and long-term consensual partnerships; Casual partners include all other types partnerships. Condom use only assessed among individuals reporting non-stable partnerships because of very low levels (<5%) condom use within stable partnerships. Those reporting non-stable partners may also have had stable partners.

Supplemental Table 4C. HIV incidence and unadjusted and adjusted incidence rate ratios comparing HIV incidence among ***male participants in HIV incident cohort (Model III)*** at each visit interval during combination HIV prevention (CHP) scale-up compared to HIV incidence in the period prior to scale-up.

Variable	*Incident cases*	*person-years*	*Incidence per 100 py (95%CI)*	*IRR (95%CI)*	*p-value*	*adjRR (95%CI)*	*p-value*
**Survey(s)**							
Pre-CHP (2-5)	109	9356	1.17 (0.96,1.4)	Ref.	-	Ref	-
Jan.2006 (6)	36	3348	1.08 (0.76,1.47)	0.92 (0.63,1.34)	0.661	0.90 (0.61,1.33)	0.603
Oct.2007 (7)	40	3791	1.06 (0.76,1.42)	0.90 (0.63,1.29)	0.572	0.89 (0.62,1.30)	0.558
Jul.2009 (8)	58	4591	1.26 (0.97,1.62)	1.08 (0.78,1.48)	0.641	1.02 (0.73,1.42)	0.926
Jan.2011 (9)	44	4497	0.98 (0.72,1.30)	0.83 (0.59,1.18)	0.309	0.92 (0.63,1.33)	0.639
Jun.2012 (10)	36	4765	0.76 (0.53,1.03)	0.64 (0.44,0.94)	0.022	0.70 (0.47,1.05)	0.083
Jul.2014 (11)	32	6069	0.53 (0.37,0.73)	0.45 (0.30,0.67)	<0.001	0.54 (0.36,0.83)	0.005
Jan.2016 (12)	27	5904	0.46 (0.31,0.65)	0.39 (0.25,0.59)	<0.001	0.46 (0.29,0.73)	0.001
**Age (years)**							
15-19	13	5894	0.22 (0.12,0.36)	0.24 (0.14,0.44)	<0.001	0.48 (0.26,0.89)	0.021
20-24	74	8203	0.90 (0.71,1.12)	Ref.	-	Ref	-
25-29	118	7965	1.48 (1.23,1.77)	1.64 (1.23,2.19)	0.001	1.32 (0.93,1.86)	0.12
30-34	91	7230	1.26 (1.02,1.54)	1.39 (1.02,1.89)	0.036	1.13 (0.78,1.63)	0.516
35-39	46	5773	0.80 (0.59,1.05)	0.88 (0.61,1.27)	0.494	0.78 (0.51,1.20)	0.254
>39 years	40	7254	0.55 (0.40,0.74)	0.61 (0.41,0.89)	0.012	0.56 (0.35,0.88)	0.013
**Male circumcision status**							
No	283	25893	1.09 (0.97,1.23)	Ref.	-	Ref	-
Yes	97	16256	0.60 (0.49,0.72)	0.54 (0.43,0.69)	<0.001	0.62 (0.48,0.79)	<0.001
**Education**							
None	12	1321	0.91 (0.49,1.52)	0.86 (0.48,1.55)	0.622	0.81 (0.45,1.46)	0.479
Primary	627	58319	1.08 (0.99,1.16)	Ref.	-	Ref	-
Secondary	216	24885	0.87 (0.76,0.99)	0.81 (0.69,0.94)	0.007	0.86 (0.73,1.01)	0.059
Tertiary/Technical school	28	6565	0.43 (0.29,0.60)	0.40 (0.27,0.58)	<0.001	0.40 (0.27,0.59)	<0.001
**Number of sex partners in the last year**							
None	13	6383	0.20 (0.11,0.34)	0.29 (0.16,0.5)	0	0.41 (0.22,0.76)	0.005

One	145	20281	0.71 (0.60,0.84)	Ref.	-	Ref	-
Two	133	10553	1.26 (1.06,1.49)	1.77 (1.39,2.24)	0	1.43 (1.03,1.99)	0.032
Three or more	89	4568	1.95 (1.57,2.38)	2.73 (2.10,3.57)	0	1.90 (1.30,2.78)	0.001
**Sex with partners outside of the community in last year**							
No	210	24560	0.86 (0.74,0.98)	Ref.	-	Ref	-
Yes	172	17760	0.97 (0.83,1.12)	1.14 (0.93,1.39)	0.218	1.07 (0.84,1.38)	0.573
**Self-reported genital ulcer disease in the last year**							
No	303	39034	0.78 (0.69,0.87)	Ref.	-	Ref	-
Yes	79	3268	2.42 (1.92,2.99)	3.13 (2.44,4.01)	<0.001	2.12 (1.64,2.73)	<0.001
**Non-marital relationships and consistent condom use***							
Stable partners only	141	21965	0.64 (0.54,0.75)	Ref.	-	Ref	-
Non-stable partners, inconsistent condom use	170	11918	1.43 (1.22,1.65)	2.23 (1.78,2.8)	<0.001	1.30 (0.89,1.88)	0.169
Non-stable partners, consistent condom use	71	8437	0.84 (0.66,1.05)	1.32 (0.99,1.76)	0.059	0.88 (0.6,1.29)	0.503
**Community type**							
Agrarian	323	36386	0.89 (0.79,0.99)	Ref.	-	Ref	-
Trading	59	5933	0.99 (0.76,1.27)	1.12 (0.85,1.48)	0.419	0.72 (0.51,1.01)	0.057
**Community HIV prevalence**							
0.4-10%	51	8063	0.63 (0.47,0.82)	Ref.	-	Ref	-
10-13%	138	17610	0.78 (0.66,0.92)	1.24 (0.9,1.70)	0.196	1.55 (1.11,2.16)	0.01
13-15%	87	9058	0.96 (0.77,1.18)	1.52 (1.07,2.14)	0.018	1.89 (1.32,2.70)	<0.001
15-20%	76	5960	1.28 (1.01,1.58)	2.01 (1.41,2.87)	<0.001	2.31 (1.59,3.36)	<0.001
>20%	30	1629	1.84 (1.26,2.58)	2.91 (1.85,4.56)	<0.001	4.11 (2.40,7.02)	<0.001

IRR=Incidence rate ratio, adjIRR=adjusted incidence rate ratio; *Stable partners include marital unions and long-term consensual partnerships; Casual partners include all other types partnerships. Condom use only assessed among individuals reporting non-stable partnerships because of very low levels (<5%) condom use within stable partnerships. Those reporting non-stable partners may also have had stable partners.

Supplemental Table 4D. HIV incidence and unadjusted and adjusted incidence rate ratios comparing HIV incidence among ***uncircumcised male participants in HIV incident cohort (Model IV)*** at each visit interval during combination HIV prevention (CHP) scale-up relative to HIV incidence in the full period prior to scale-up.

Variable	*Incident cases*	*person-years*	*Incidence per 100 py (95%CI)*	*IRR (95%CI)*	*p-value*	*adjRR (95%CI)*	*p-value*
**Survey(s)**							
Pre-CHP (2-5)	94	7773	1.21 (0.98,1.47)	Ref.	-	Ref	-
Jan.2006 (6)	30	2456	1.22 (0.83,1.71)	1.01 (0.67,1.52)	0.98	0.96 (0.63,1.46)	0.851
Oct.2007 (7)	31	2590	1.20 (0.82,1.67)	0.98 (0.66,1.48)	0.939	0.92 (0.61,1.40)	0.701
Jul.2009 (8)	40	2927	1.37 (0.99,1.83)	1.12 (0.78,1.63)	0.535	1.00 (0.68,1.47)	0.988
Jan.2011 (9)	32	2571	1.24 (0.86,1.73)	1.02 (0.68,1.53)	0.918	1.00 (0.67,1.51)	0.984
Jun.2012 (10)	24	2493	0.96 (0.63,1.40)	0.79 (0.50,1.24)	0.303	0.77 (0.49,1.22)	0.266
Jul.2014 (11)	17	2779	0.61 (0.36,0.95)	0.50 (0.30,0.84)	0.009	0.46 (0.26,0.81)	0.007
Jan.2016 (12)	15	2303	0.65 (0.37,1.04)	0.53 (0.31,0.92)	0.024	0.51 (0.29,0.88)	0.016
**Age (years)**							
15-19	10	3736	0.27 (0.13,0.47)	0.22 (0.11,0.43)	<0.001	0.45 (0.22,0.92)	0.029
20-24	61	5082	1.20 (0.92,1.53)	Ref.	-	Ref	-
25-29	89	4765	1.87 (1.51,2.28)	1.55 (1.12,2.15)	0.008	1.35 (0.92,1.97)	0.125
30-34	60	4403	1.36 (1.05,1.74)	1.13 (0.79,1.61)	0.506	1.00 (0.65,1.52)	0.989
35-39	33	3426	0.96 (0.67,1.33)	0.80 (0.52,1.22)	0.296	0.77 (0.47,1.25)	0.286
>39 years	30	4482	0.67 (0.46,0.94)	0.55 (0.36,0.86)	0.008	0.61 (0.36,1.02)	0.060
**Education**							
None	11	890	1.24 (0.64,2.12)	0.96 (0.52,1.76)	0.893	0.96 (0.52,1.81)	0.911
Primary	217	16852	1.29 (1.12,1.47)	Ref.	-	Ref	-
Secondary	45	6055	0.74 (0.55,0.98)	0.58 (0.42,0.80)	0.001	0.68 (0.49,0.94)	0.021
Tertiary/Technical school	10	2017	0.50 (0.25,0.87)	0.38 (0.20,0.73)	0.003	0.38 (0.20,0.71)	0.003
**Number of sex partners in the last year**							
None	8	4028	0.20 (0.09,0.37)	0.23 (0.11,0.48)	<0.001	0.30 (0.14,0.65)	0.002
One	108	12602	0.86 (0.71,1.03)	Ref.	-	Ref	-
Two	100	6284	1.59 (1.30,1.92)	1.86 (1.42,2.45)	<0.001	1.40 (0.96,2.03)	0.080
Three or more	65	2723	2.39 (1.85,3.02)	2.80 (2.05,3.81)	<0.001	1.64 (1.07,2.51)	0.023

**Sex with partners outside of the community in last year**							
No	155	15004	1.03 (0.88,1.20)	Ref.	-	Ref	-
Yes	128	10889	1.18 (0.98,1.39)	1.14 (0.90,1.45)	0.267	1.05 (0.80,1.39)	0.71
**Self-reported genital ulcer disease in the last year**							
No	211	23630	0.89 (0.78,1.02)	Ref.	-	Ref	-
Yes	72	2249	3.20 (2.52,4.00)	3.61 (2.75,4.72)	<0.001	2.49 (1.89,3.3)	<0.001
**Non-marital relationships and consistent condom use***							
Stable partners only	97	13492	0.72 (0.59,0.87)	Ref.	-	Ref	-
Non-stable partners, inconsistent condom use	136	7180	1.89 (1.59,2.23)	2.65 (2.04,3.44)	<0.001	1.54 (1.00,2.36)	0.048
Non-stable partners, consistent condom use	50	5222	0.96 (0.72,1.25)	1.34 (0.95,1.89)	0.094	0.90 (0.57,1.40)	0.631
**Community type**							
Agrarian	245	22779	1.08 (0.95,1.22)	Ref.	-	Ref	-
Trading	38	3114	1.22 (0.87,1.65)	1.14 (0.81,1.6)	0.461	0.75 (0.51,1.11)	0.155
**Community HIV prevalence**							
0.4-10%	34	5350	0.64 (0.45,0.87)	Ref.	-	Ref	-
10-13%	106	10250	1.03 (0.85,1.24)	1.62 (1.10,2.39)	0.014	1.90 (1.27,2.84)	0.002
13-15%	73	5684	1.28 (1.01,1.60)	2.02 (1.34,3.03)	0.001	2.41 (1.58,3.66)	<0.001
15-20%	55	3782	1.45 (1.10,1.87)	2.28 (1.49,3.50)	<0.001	2.70 (1.74,4.19)	<0.001
>20%	15	827	1.81 (1.04,2.89)	2.85 (1.55,5.24)	0.001	3.78 (1.90,7.53)	<0.001

IRR=Incidence rate ratio, adjIRR=adjusted incidence rate ratio; *Stable partners include marital unions and long-term consensual partnerships; Casual partners include all other types partnerships. Condom use only assessed among individuals reporting non-stable partnerships because of very low levels (<5%) condom use within stable partnerships. Those reporting non-stable partners may also have had stable partners.

Supplemental Table 4E. HIV incidence and unadjusted and adjusted incidence rate ratios comparing HIV incidence among ***circumcised male participants in HIV incident cohort (Model V)*** at each visit interval during combination HIV prevention (CHP) scale-up relative to HIV incidence in the full period prior to scale-up.

Variable	*Incident cases*	*person-years*	*Incidence per 100 py (95%CI)*	*IRR (95%CI)*	*p-value*	*adjRR (95%CI)*	*p-value*
**Survey(s)**							
Pre-CHP (2-5)	15	1570	0.96 (0.55,1.52)	Ref.	-	Ref	-
Jan.2006 (6)	4	748	0.53 (0.17,1.24)	0.56 (0.19,1.68)	0.301	0.61 (0.2,1.86)	0.385
Oct.2007 (7)	9	1196	0.75 (0.36,1.36)	0.79 (0.34,1.79)	0.568	0.73 (0.32,1.66)	0.449
Jul.2009 (8)	18	1662	1.08 (0.66,1.66)	1.13 (0.57,2.24)	0.728	0.95 (0.46,1.97)	0.892
Jan.2011 (9)	12	1926	0.62 (0.33,1.04)	0.65 (0.30,1.38)	0.263	0.70 (0.31,1.58)	0.392
Jun.2012 (10)	12	2268	0.53 (0.28,0.89)	0.55 (0.26,1.18)	0.123	0.56 (0.24,1.31)	0.180
Jul.2014 (11)	15	3288	0.46 (0.26,0.73)	0.47 (0.23,0.97)	0.041	0.65 (0.31,1.39)	0.269
Jan.2016 (12)	12	3599	0.33 (0.18,0.56)	0.35 (0.16,0.74)	0.006	0.43 (0.19,0.99)	0.047
**Age (years)**							
15-19	3	2125	0.14 (0.04,0.37)	0.33 (0.1,1.17)	0.087	0.56 (0.15,2.11)	0.387
20-24	13	3081	0.42 (0.23,0.69)	Ref.	-	Ref	-
25-29	28	3159	0.89 (0.60,1.26)	2.10 (1.09,4.05)	0.027	1.32 (0.58,3.02)	0.505
30-34	31	2793	1.11 (0.76,1.55)	2.63 (1.37,5.02)	0.004	1.67 (0.74,3.77)	0.220
35-39	13	2336	0.56 (0.31,0.92)	1.31 (0.61,2.84)	0.488	0.87 (0.34,2.21)	0.762
>39 years	9	2763	0.33 (0.16,0.59)	0.77 (0.33,1.80)	0.541	0.42 (0.15,1.20)	0.105
**Education**							
None	36	7640	0.47 (0.33,0.64)	Ref.	-	Ref	-
Primary	32	4240	0.75 (0.52,1.05)	1.61 (1.00,2.59)	0.052	1.53 (0.76,3.07)	0.233
Secondary	21	4439	0.47 (0.30,0.71)	0.72 (0.44,1.17)	0.186	0.80 (0.49,1.32)	0.382
Tertiary/Technical school		1098	0.55 (0.22,1.11)	0.83 (0.36,1.92)	0.661	0.83 (0.35,1.99)	0.684
**Number of sex partners in the last year**							
None	5	2274	0.22 (0.08,0.47)	0.47 (0.18,1.19)	0.112	0.86 (0.26,2.78)	0.795
One	36	7640	0.47 (0.33,0.64)	Ref.	-	Ref	-
Two	32	4240	0.75 (0.52,1.05)	1.61 (1.00,2.59)	0.052	1.53 (0.76,3.07)	0.233
Three or more	24	1825	1.31 (0.86,1.91)	2.80 (1.67,4.70)	<0.001	2.77 (1.17,6.52)	0.020

**Sex with partners outside of the community in last year**							
No	53	9455	0.56 (0.42,0.73)	Ref.	-	Ref	-
Yes	44	6801	0.65 (0.47,0.86)	1.16 (0.77,1.72)	0.479	1.14 (0.67,1.95)	0.630
**Self-reported genital ulcer disease in the last year**							
No	90	15240	0.59 (0.48,0.72)	Ref.	-	Ref	-
Yes	7	1015	0.69 (0.30,1.33)	1.17 (0.54,2.53)	0.687	0.77 (0.34,1.76)	0.539
**Non-marital relationships and consistent condom use***							
Stable partners only	42	8392	0.50 (0.36,0.67)	Ref.	-	Ref	-
Non-stable partners, inconsistent condom use	34	4674	0.73 (0.51,1.00)	1.46 (0.93,2.30)	0.102	0.76 (0.35,1.62)	0.473
Non-stable partners, consistent condom use	21	3190	0.66 (0.42,0.98)	1.32 (0.78,2.23)	0.301	0.82 (0.39,1.73)	0.602
**Community type**							
Agrarian	76	13445	0.57 (0.45,0.70)	Ref.	-	Ref	-
Trading	21	2811	0.75 (0.47,1.11)	1.32 (0.82,2.14)	0.258	0.68 (0.35,1.32)	0.253
**Community HIV prevalence**							
0.4-10%	17	2666	0.64 (0.38,0.99)	Ref.	-	Ref	-
10-13%	32	7295	0.44 (0.30,0.61)	0.69 (0.38,1.24)	0.212	0.84 (0.46,1.55)	0.578
13-15%	14	3369	0.42 (0.23,0.67)	0.65 (0.32,1.32)	0.234	0.83 (0.38,1.82)	0.645
15-20%	19	2124	0.89 (0.55,1.36)	1.40 (0.73,2.70)	0.314	1.53 (0.70,3.32)	0.283
>20%	15	802	1.87 (1.08,2.98)	2.93 (1.46,5.86)	0.002	3.93 (1.60,9.62)	0.003

IRR=Incidence rate ratio, adjIRR=adjusted incidence rate ratio; *Stable partners include marital unions and long term consensual partnerships; Casual partners include all other types partnerships. Condom use only assessed among individuals reporting non-stable partnerships because of very low levels (<5%) condom use within stable partnerships. Those reporting non-stable partners may also have had stable partners.

Supplemental Table 5. HIV incidence and unadjusted and adjusted incidence rate ratios comparing HIV incidence ***among individuals 20 years and older (Model VI)*** in each visit interval during combination HIV prevention (CHP) scale-up compared to HIV incidence in the period prior to scale-up.

Survey(s)	*HIV incidence per 100 py (95%CI)*	*IRR (95%CI)*	*p-value*	*adjIRR (95% CI)*	*p-value*
Pre-CHP (2-5)	1.24 (1.09,1.41)	Ref.	-	Ref	-
Jan.2006 (6)	1.09 (0.87,1.36)	0.88 (0.68,1.14)	0.326	0.88 (0.68,1.14)	0.335
Oct.2007 (7)	1.25 (1.02,1.51)	1.00 (0.79,1.27)	0.981	0.99 (0.78,1.26)	0.948
Jul.2009 (8)	1.31 (1.09,1.56)	1.05 (0.85,1.31)	0.641	0.95 (0.76,1.20)	0.678
Jan.2011 (9)	1.17 (0.95,1.41)	0.94 (0.74,1.18)	0.576	0.95 (0.74,1.21)	0.663
Jun.2012 (10)	0.88 (0.70,1.09)	0.71 (0.55,0.91)	0.007	0.71 (0.54,0.92)	0.011
Jul.2014 (11)	0.70 (0.56,0.86)	0.56 (0.44,0.72)	<0.001	0.60 (0.46,0.79)	<0.001
Jan.2016 (12)	0.72 (0.58,0.89)	0.58 (0.45,0.75)	<0.001	0.60 (0.46,0.79)	<0.001

Supplemental Table 6. HIV incidence and unadjusted and adjusted incidence rate ratios comparing HIV incidence ***among sexually active persons only*** in each visit interval during combination HIV prevention (CHP) scale-up compared to HIV incidence in the period prior to scale-up.

Survey(s)	*HIV incidence per 100 py (95%CI)*	*IRR (95%CI)*	*p-value*	*adjIRR (95% CI)*	*p-value*
Pre-CHP (2-5)	1.25 (1.10,1.42)	Ref.	-	Ref	-
Jan.2006 (6)	1.22 (0.98,1.51)	0.98 (0.76,1.26)	0.860	0.96 (0.75,1.25)	0.784
Oct.2007 (7)	1.30 (1.06,1.57)	1.04 (0.82,1.31)	0.756	1.02 (0.80,1.30)	0.859
Jul.2009 (8)	1.33 (1.10,1.59)	1.06 (0.85,1.33)	0.593	0.96 (0.76,1.21)	0.704
Jan.2011 (9)	1.17 (0.95,1.42)	0.93 (0.74,1.18)	0.560	0.95 (0.74,1.21)	0.668
Jun.2012 (10)	0.91 (0.73,1.13)	0.73 (0.56,0.94)	0.014	0.71 (0.55,0.93)	0.014
Jul.2014 (11)	0.75 (0.60,0.92)	0.60 (0.47,0.77)	<0.001	0.63 (0.48,0.82)	0.001
Jan.2016 (12)	0.75 (0.60,0.93)	0.60 (0.47,0.77)	<0.001	0.62 (0.47,0.81)	<0.001

Supplemental Table 7: HIV incidence and unadjusted and adjusted incidence rate ratios with in each visit interval during combination HIV prevention (CHP) scale-up compared to HIV incidence in the period prior to scale-up. Final model was also adjusted for participants' ***birth cohort (Model VIII)***.

Survey(s)	*HIV incidence per 100 py (95%CI)*	*IRR (95%CI)*	*p-value*	*adjIRR (95% CI)*	*p-value*
Pre-CHP (2-5)	1.17 (1.03,1.32)	Ref.	-	Ref.	-
Jan.2006 (6)	1.11 (0.89,1.36)	0.95 (0.74,1.21)	0.659	0.89 (0.69,1.16)	0.391
Oct.2007 (7)	1.20 (0.98,1.44)	1.02 (0.82,1.29)	0.839	0.92 (0.71,1.19)	0.535
Jul.2009 (8)	1.23 (1.02,1.45)	1.05 (0.85,1.30)	0.670	0.84 (0.63,1.12)	0.236
Jan.2011 (9)	1.07 (0.88,1.29)	0.91 (0.73,1.15)	0.436	0.81 (0.59,1.10)	0.181
Jun.2012 (10)	0.83 (0.67,1.02)	0.71 (0.55,0.91)	0.006	0.60 (0.42,0.86)	0.005
Jul.2014 (11)	0.66 (0.53,0.81)	0.56 (0.44,0.72)	<0.001	0.49 (0.32,0.73)	0.001
Jan.2016 (12)	0.66 (0.53,0.81)	0.56 (0.44,0.72)	<0.001	0.46 (0.29,0.72)	0.001

Supplemental Table 8: ***Inverse probability weighted (Model IX)*** unadjusted and adjusted incidence rate ratios within each visit interval during combination HIV prevention (CHP) scale-up compared to HIV incidence in the period prior to scale-up.

Survey(s)	*IRR (95%CI)*	*p-value*	*adjIRR (95% CI)*	*p-value*
Pre-CHP (2-5)	Ref.	-	Ref.	-
Jan.2006 (6)	0.95 (0.73,1.23)	0.704	0.94 (0.72,1.22)	0.658
Oct.2007 (7)	1.09 (0.85,1.37)	0.496	1.01 (0.79,1.28)	0.942
Jul.2009 (8)	1.08 (0.85,1.35)	0.528	0.95 (0.75,1.19)	0.654
Jan.2011 (9)	0.98 (0.77,1.24)	0.862	0.99 (0.77,1.26)	0.953
Jun.2012 (10)	0.73 (0.56,0.94)	0.019	0.75 (0.58,0.98)	0.037
Jul.2014 (11)	0.61 (0.47,0.78)	<0.001	0.64 (0.49,0.84)	0.002
Jan.2016 (12)	0.56 (0.42,0.73)	<0.001	0.58 (0.44,0.77)	<0.001

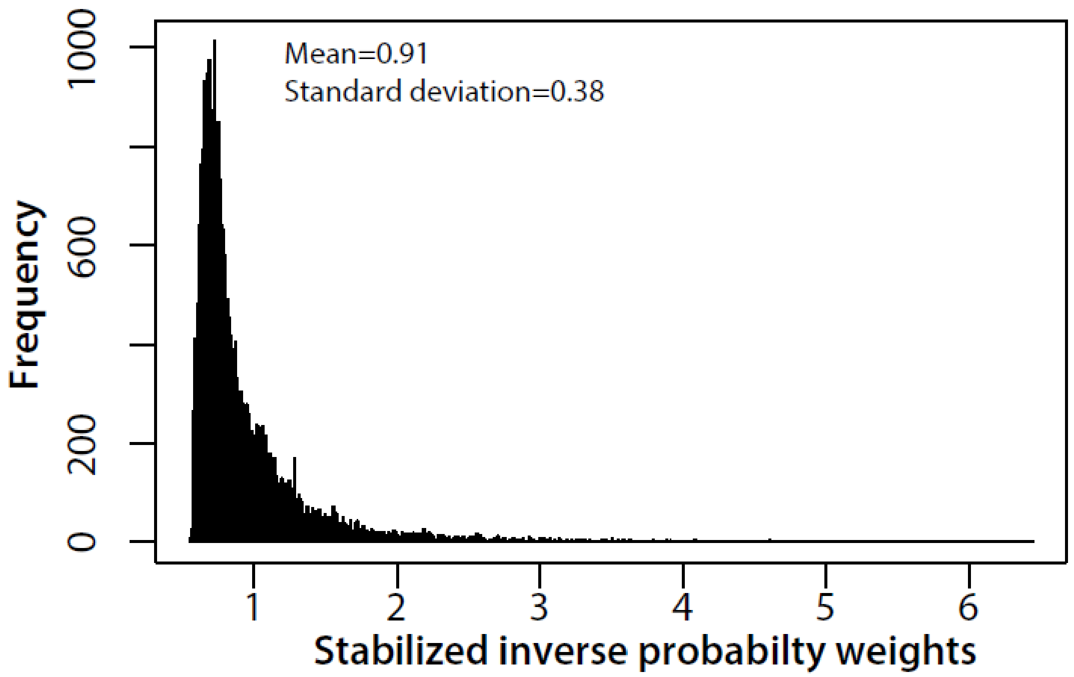

Supplemental Figure 5. Histogram of stablized inverse probablity weights among HIV-negative persons in HIV incidence cohort.

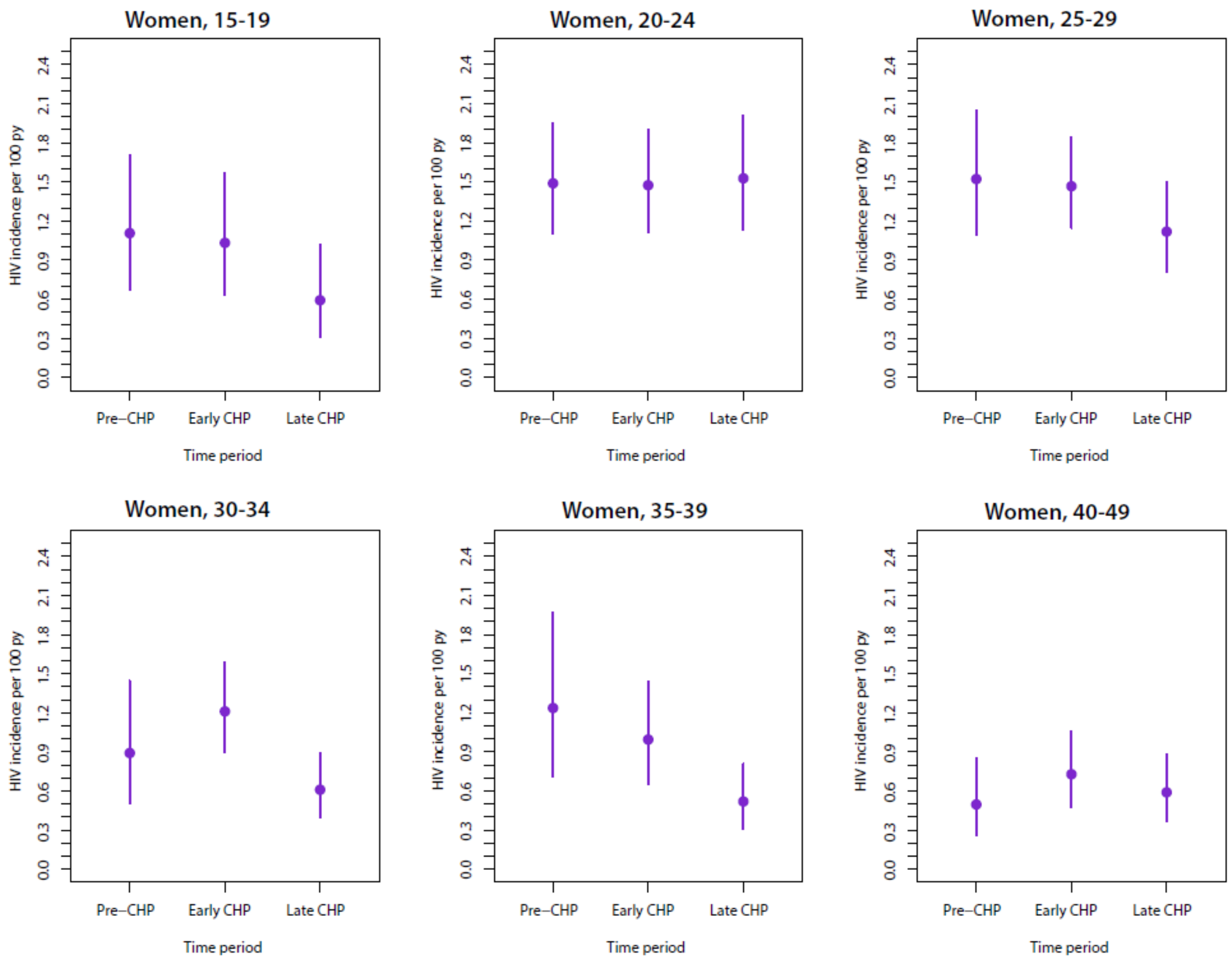

Supplemental Figure 6A. HIV incidence among women by age-group prior to CHP scale-up (visits 2-5), during early CHP scale-up (visits 6-9), and in later phases of CHP scale-up (visit 10-12).

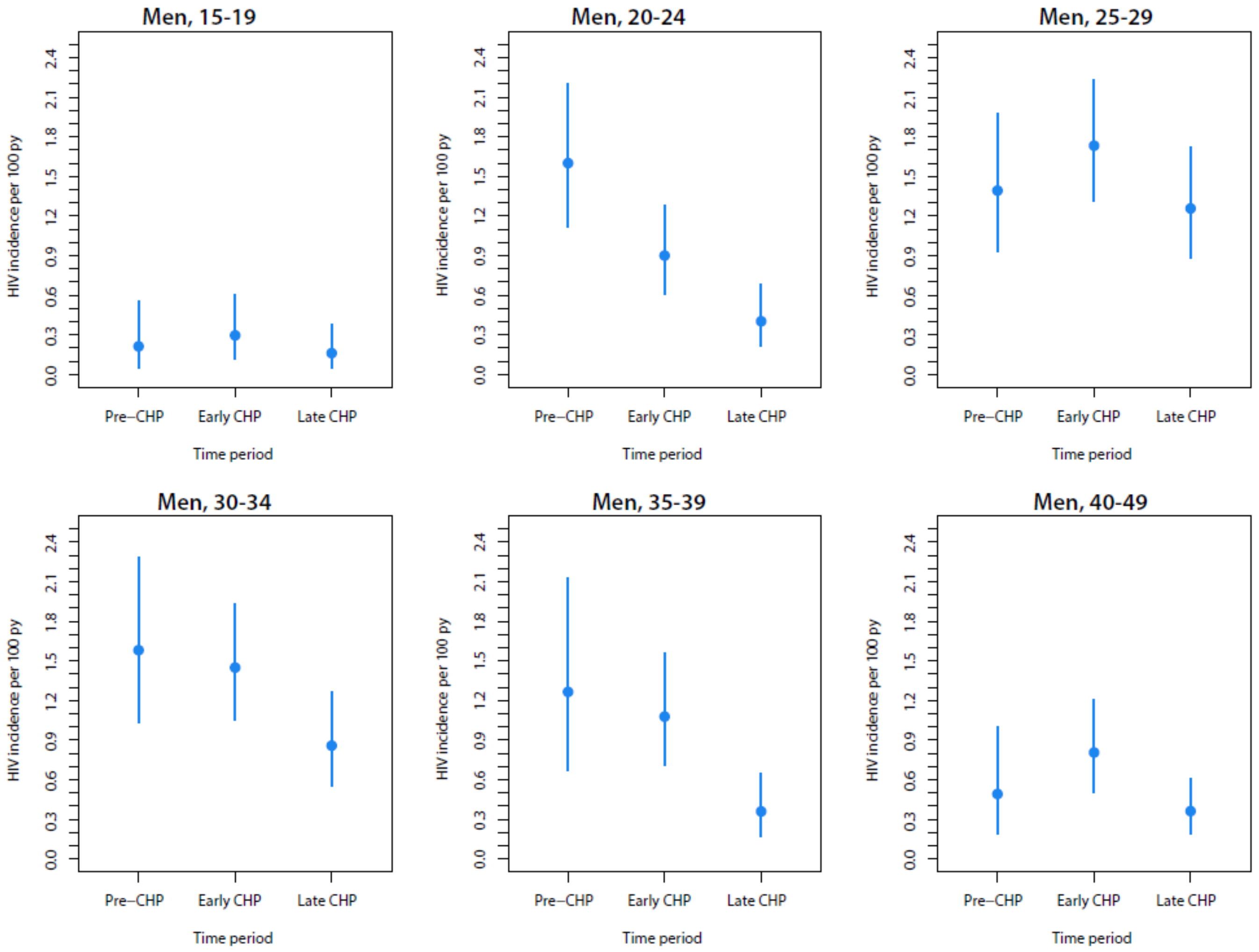

Supplemental Figure 6B. HIV incidence among men by age-group prior to CHP scale-up (visits 2-5), during early CHP scale-up (visits 6-9), and in later phases of CHP scale-up (visit 10-12).

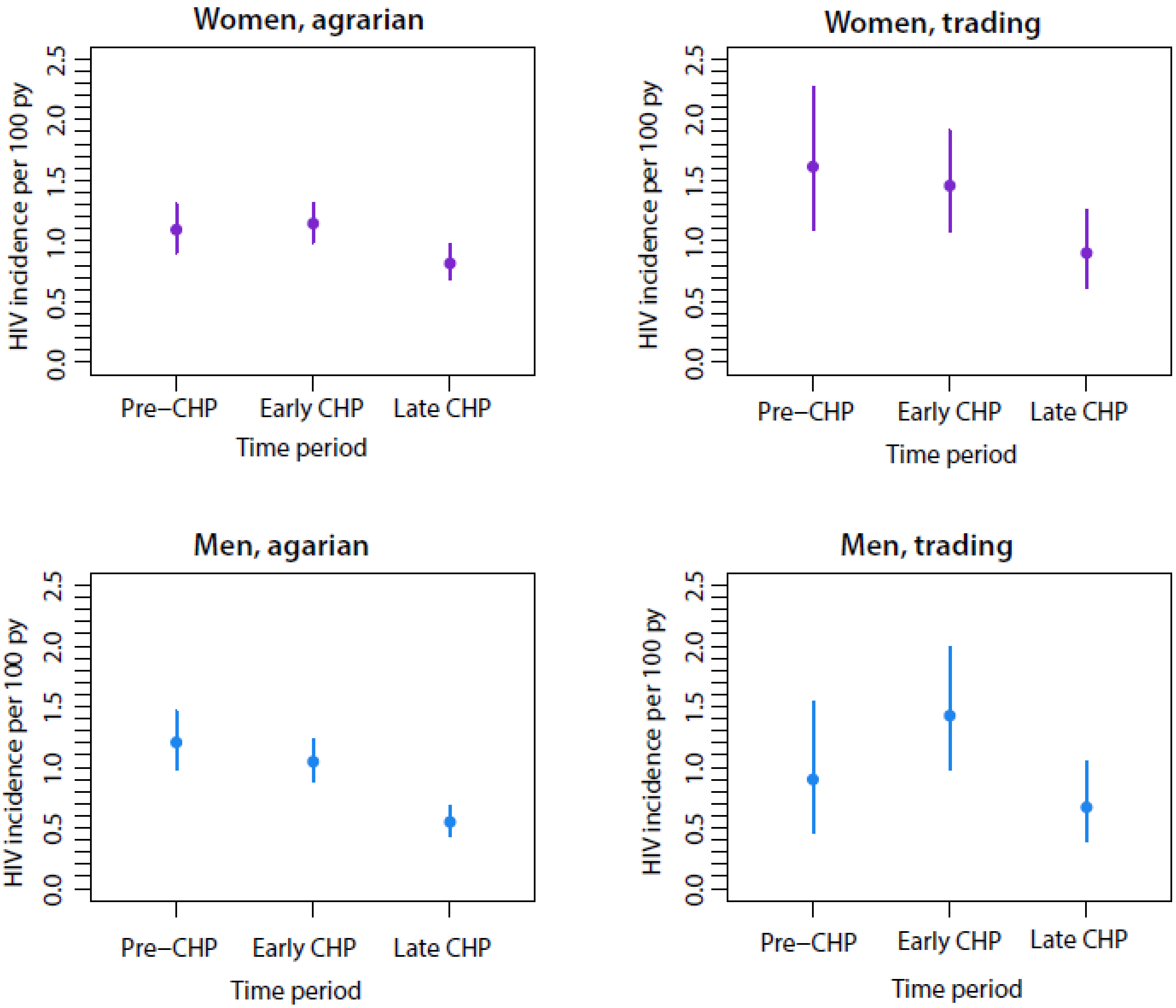

Supplemental Figure 6C. HIV incidence by gender and community-type prior to CHP scale-up (visits 2-5), during early CHP scale-up (visits 6-9), and in later phases of CHP scale-up (visit 10-12).

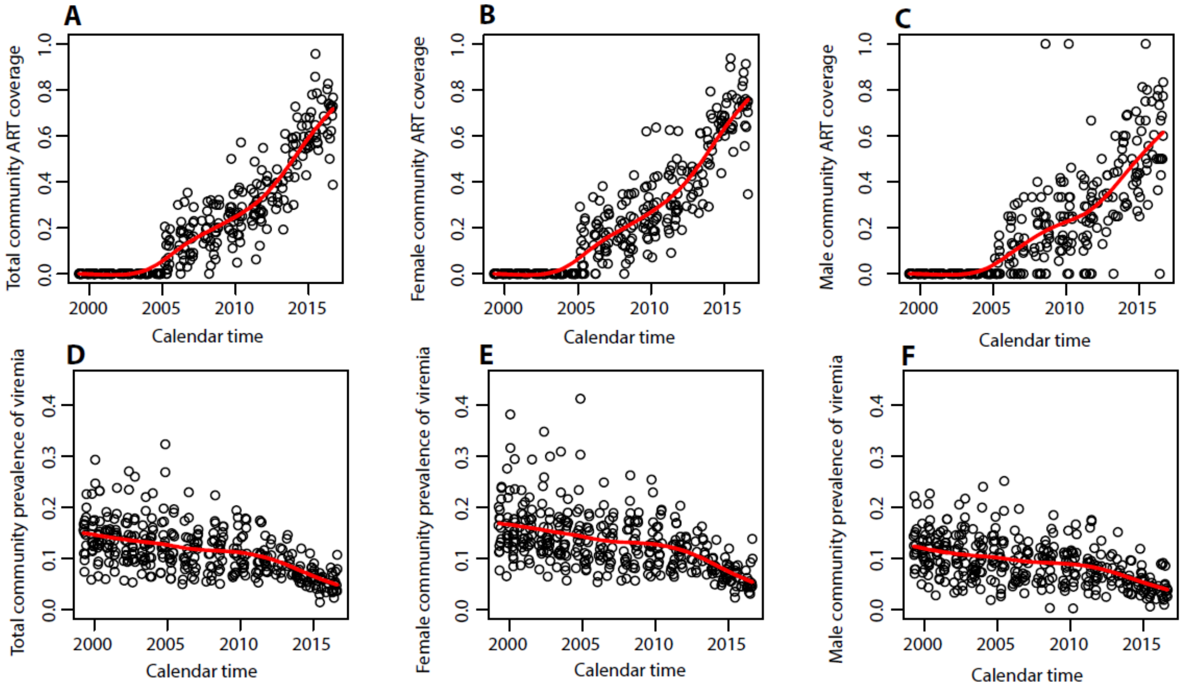

Supplementary Figure 7. Scale-up of ART and declining population viremia in RCCS communities over calendar time. Each circle represents ART coverage or prevalence of HIV viremia in an RCCS community at one of the 12 survey visits. Data are plotted at the median interview date for that community at that visit. Smoothing splines (shown in red) were fitted to the data. **A)** Community-level ART coverage in men and women. **B)** Community-level ART coverage in women. **C)** Community-level ART coverage in men. **D)** Community prevalence of HIV viremia. **E)** Community female prevalence of viremia. **F)** Community male prevalence of viremia.

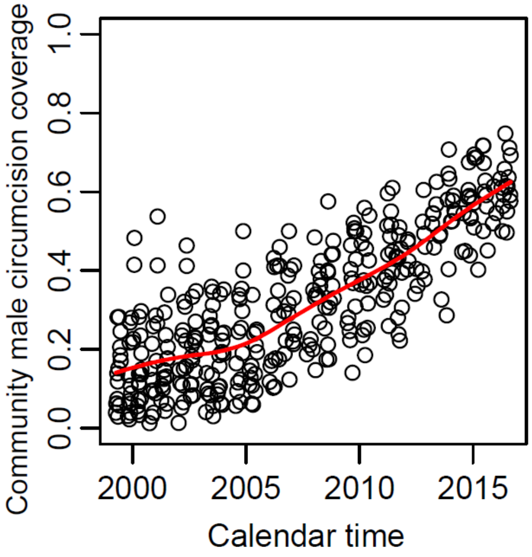

Supplementary Figure 8. Scale-up of male circumcision (MC) coverage in RCCS communities over calendar time. Each circle represents MC coverage in an RCCS community at one of the 12 survey visits. Data are plotted at the median interview date for that community. A smoothing spline (shown in red) was fitted to the data.

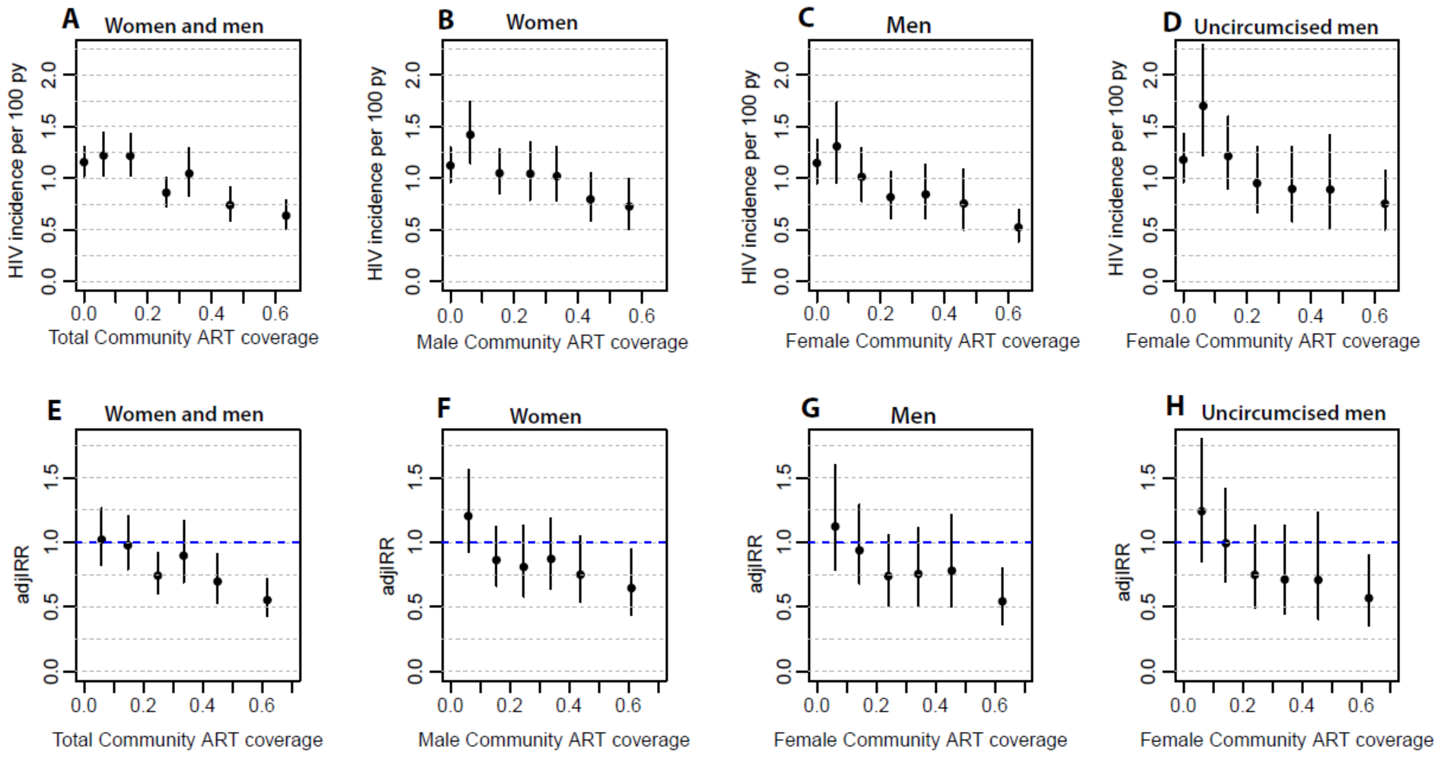

Supplementary Figure 9. HIV incidence and individual HIV-risk as a function of community ART coverage in the total population, women, men, and uncircumcised men. Data are plotted at the median coverage-level within each community-level ART coverage category (0-10%, 10-20%, 20-30%, 30-40%, 40-50%, >50%). The reference group for the incidence rate ratios is community ART coverage levels of 0-10%. Models were adjusted for potential confounders as outlined for models 1-IV in supplementary appendix excluding survey visit.

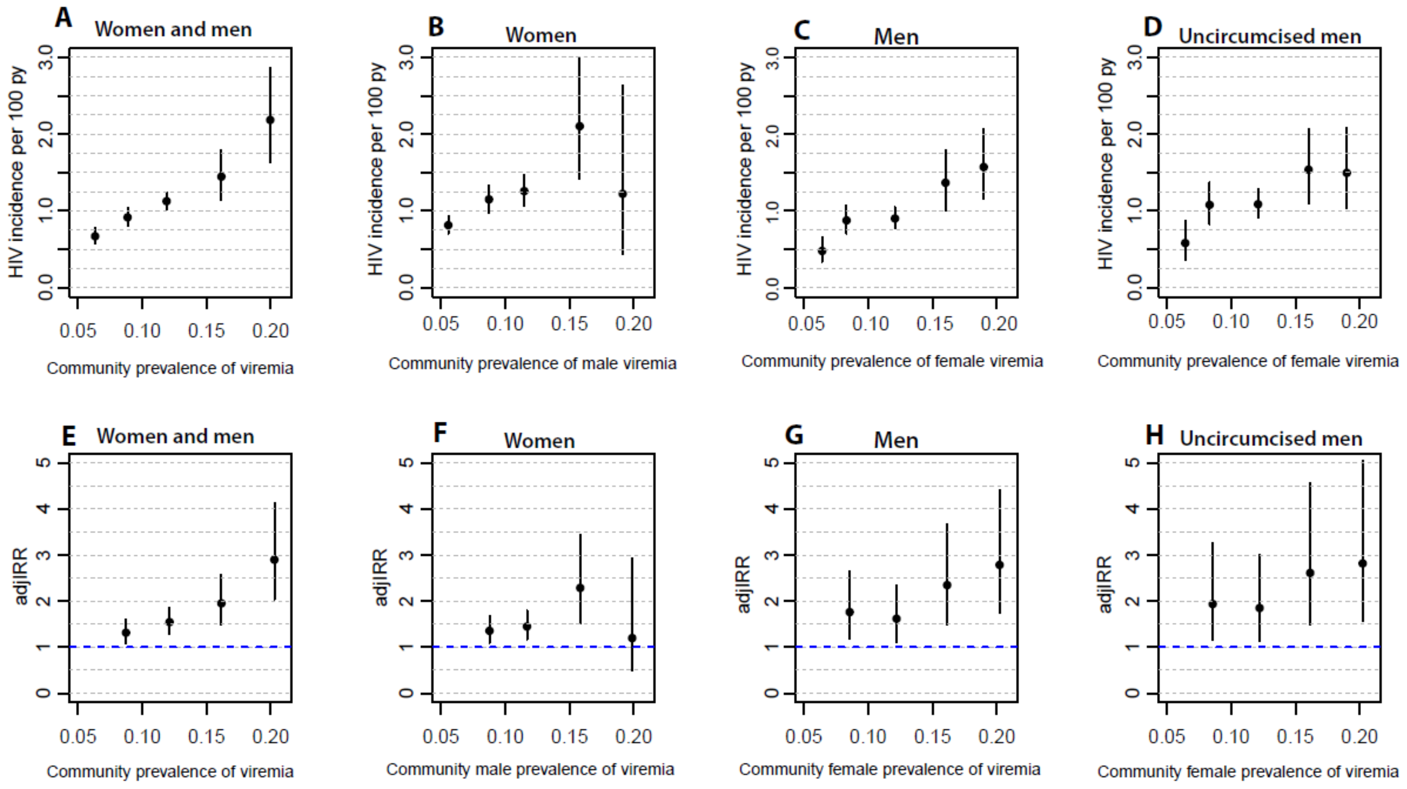

Supplementary Figure 10. HIV incidence and individual HIV-risk as a function of community prevalence of HIV viremia in the total population, women, men, and uncircumcised men. Data are plotted at the median prevalence within each community-level prevalence category (0-7.5%, 7.5-10%, 10-12.5%, 12.5-15%, 15-17.5%, >17.5%) The reference group for the incidence rate ratios was 0-7.5%. Models were adjusted for potential confounders as outlined for models 1-IV in supplementary appendix excluding survey visit.

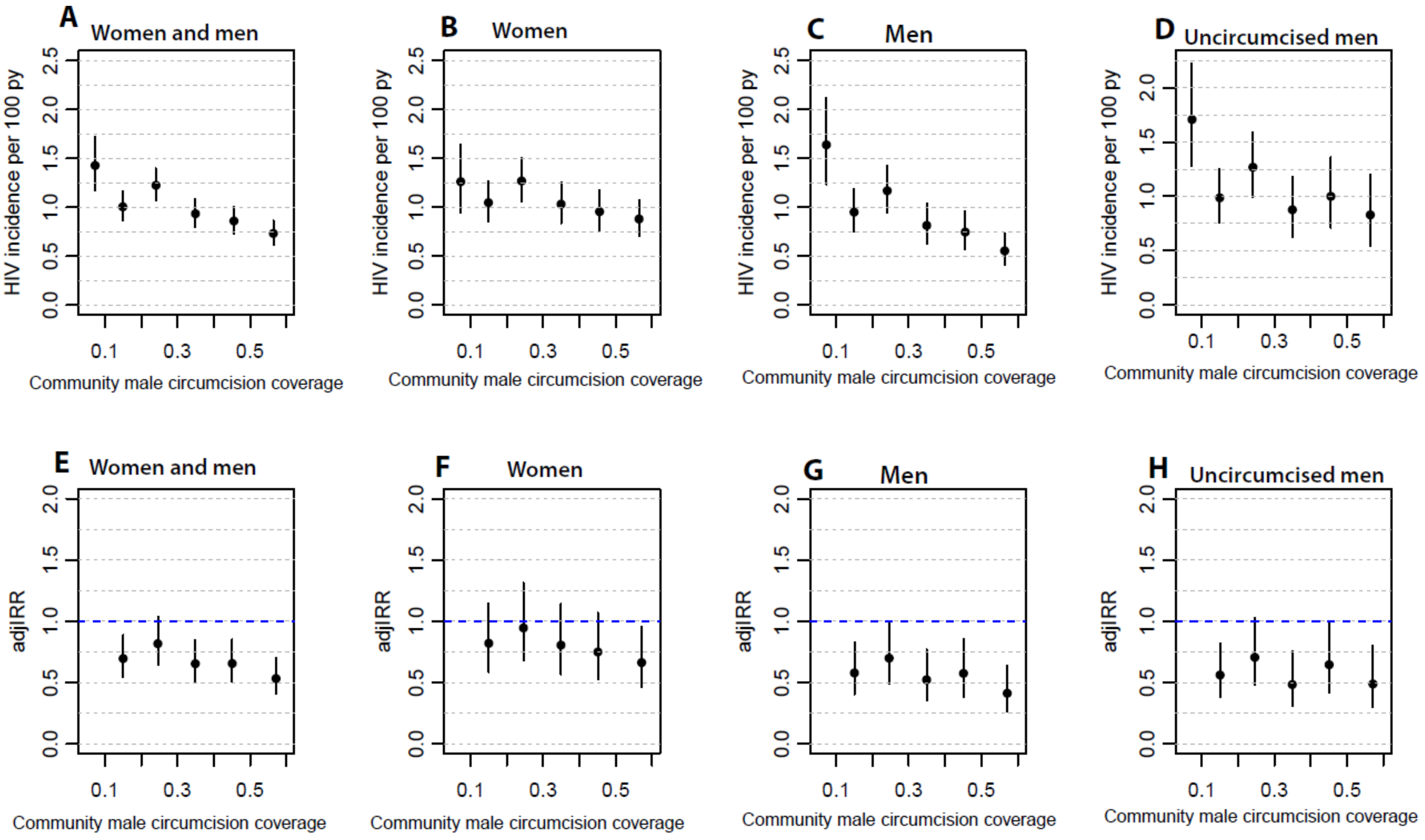

Supplementary Figure 11. HIV incidence and individual HIV-risk as a function of community MC coverage in the total population, women, men, and uncircumcised men. Data are plotted at the median coverage-level within each community-level MC coverage category (0-10%, 10-20%, 20-30%, 30-40%, 40-50%, >50%). The reference group for the incidence rate ratios is community ART coverage levels of 0-10%. Models were adjusted for potential confounders as outlined for models 1-IV in supplementary appendix excluding survey visit.
